# Predicting cell morphological responses to perturbations using generative modeling

**DOI:** 10.1038/s41467-024-55707-8

**Published:** 2025-01-08

**Authors:** Alessandro Palma, Fabian J. Theis, Mohammad Lotfollahi

**Affiliations:** 1 Department of Computational Health, Institute of Computational Biology, Helmholtz Zentrum München, Munich Germany; 2 School of Computing, Information and Technology, Technical University of Munich, Munich, Germany; 3 TUM School of Life Sciences Weihenstephan, Technical University of Munich, Freising, Germany; 4 Wellcome Sanger Institute, Wellcome Genome Campus, Hinxton, Cambridge, UK; 5 Cambridge Stem Cell Institute, University of Cambridge, Cambridge, UK

**Keywords:** Machine learning, High-throughput screening

## Abstract

Advancements in high-throughput screenings enable the exploration of rich phenotypic readouts through high-content microscopy, expediting the development of phenotype-based drug discovery. However, analyzing large and complex high-content imaging screenings remains challenging due to incomplete sampling of perturbations and the presence of technical variations between experiments. To tackle these shortcomings, we present IMage Perturbation Autoencoder (IMPA), a generative style-transfer model predicting morphological changes of perturbations across genetic and chemical interventions. We show that IMPA accurately captures morphological and population-level changes of both seen and unseen perturbations on breast cancer and osteosarcoma cells. Additionally, IMPA accounts for batch effects and can model perturbations across various sources of technical variation, further enhancing its robustness in diverse experimental conditions. With the increasing availability of large-scale high-content imaging screens generated by academic and industrial consortia, we envision that IMPA will facilitate the analysis of microscopy data and enable efficient experimental design via in-silico perturbation prediction.

## Abstract (web summary)

Predicting morphological impacts of perturbations using computational methods can aid treatment discovery. Here, authors present IMPA, a generative model that predicts control cell responses to measured and unmeasured chemical and genetic perturbations conditioned on an interpretable perturbation space.

## Introduction

The advent of high-throughput microscopy-based phenotypic profiling has made it possible to screen thousands of chemical^1–4^ and genetic^5,6^ perturbations in parallel to identify potential drug targets, Modes of Action (MoAs) and gene functions across diverse biological settings. Such profiling assays measure morphological changes of sub-cellular structures in selected cell lines by staining them with multiple fluorescent dyes and highlighting different organelles and components. An example is Cell Painting^7^, one of the most commonly used unbiased image-based profiling assays^8^ that measures different cellular substructures using fluorescent dyes. While current technologies are scalable, exploring the large space of potentially synthesizable drug molecules or genetic perturbations^9^ is complex. Consequently, performing larger screens can be experimentally challenging and costly, requiring the help of computational approaches^10^. In this context, models are required to predict morphological changes due to unmeasured perturbations in the experiment, facilitating experimental design by generating cellular responses that can narrow down the hypothesis space and aid in phenotype-based drug discovery. However, microscopy images are affected by technical effects deriving from samples from different experimental sources^11^. Therefore, an additional challenge for computational methods is to account for technical variability to avoid spurious feature learning and predictions confounded with batch effect.

Existing frameworks for predicting phenotype responses in high-throughput image-based data have been explored in multiple settings. Supervised tasks include drug MoA^12–14^ and toxicity^15^ prediction, and assay activity annotation^16^. Concurrently, generative models can be used to synthesize in-silico representations of cell image features under specific interventions and conditions^17^ or predict responses to drug combinations^18^. The prediction of image-based drug responses with generative modeling was already explored in the form of latent traversals of a Generative Adversarial Network (GAN)^19^ trained to generate drug-perturbed images conditionally on perturbation labels^20^. However, such a study only tackles morphological transformations caused by compounds seen during training, limiting the model’s ability to generalize to new drugs. To solve this problem, Mol2Image^21^ employs a conditional flow-based model to generate cellular images based on the molecular structure of unseen chemical perturbations. Mol2Image focuses on generating synthetic drug responses from noise and does not account for perturbation-induced morphological changes in real control cell images. Meanwhile, PhenDiff^22^ did interpret the synthetic perturbation task as the transformation of real control images into their treated counterparts. However, the authors only use chemical perturbations and not genetic interventions and do not discuss strategies to remove batch effects and avoid confounding in the treatment effect prediction. Despite advancements in predictive models, accounting for the technical variability that can obscure biological signals remains a challenge. In microscopy, generative models have been explored for correcting batch effects directly in the image domain, exploiting histopathological datasets^23^ as well as high-content screenings^24,25^. However, no previous study has focused on summarizing the batch correction and perturbation prediction objectives into the same model under a simple task shift specification.

Gaps in existing work prompt our approach to answering how an image of an untreated cell would appear if treated with a specific perturbation. To address this aspect, we introduce the IMage Perturbation Autoencoder (IMPA), a deep generative model designed to predict cellular responses to perturbations and remove batch effects in high-throughput image-based profiling screens. IMPA adopts a style transfer approach^26–28^ for the image-to-image translation^29^ task. The model learns to decompose a cell image into its style (perturbation/batch representation) and content (cell representation). Through training, IMPA can transfer a cell to a desired style (i.e., a different perturbation or batch), while preserving its style-independent content. We use our model in the synthetic perturbation setting to transform real images of unperturbed cells into their treated counterpart. In this context, IMPA stands out for its utilization of unpaired data, removing the requirement to screen images of the same cell before and after treatment. Moreover, one can use IMPA for technical effect correction by learning to transport all microscopy images to a reference batch.

We demonstrate the effectiveness of IMPA on the perturbation prediction tasks using diverse datasets, including drug screens on MCF-7 cells and a combination of CRISPR, overexpression and chemical perturbation assays on U2OS cells. IMPA learns morphological responses to treatment both at a single-cell level and on larger fields of view, improving the performance over six baselines. Moreover, we showcase the advantage of IMPA as a batch correction tool using a siRNA perturbation dataset focusing on U2OS cells. Finally, we investigate IMPA’s interpretability and flexibility in learning biologically meaningful perturbation spaces, generalizing to perturbations not seen during the training process and detecting subtle phenotypic effects in complex microscopy datasets. Our model enables efficient target discovery in phenotypic screens, streamlining experimental design and enhancing our understanding of morphological alterations in high-throughput image-based profiling.

## Results

### Learning morphological responses to perturbations and technical effect shifts using style transfer

**Fig. 1:**
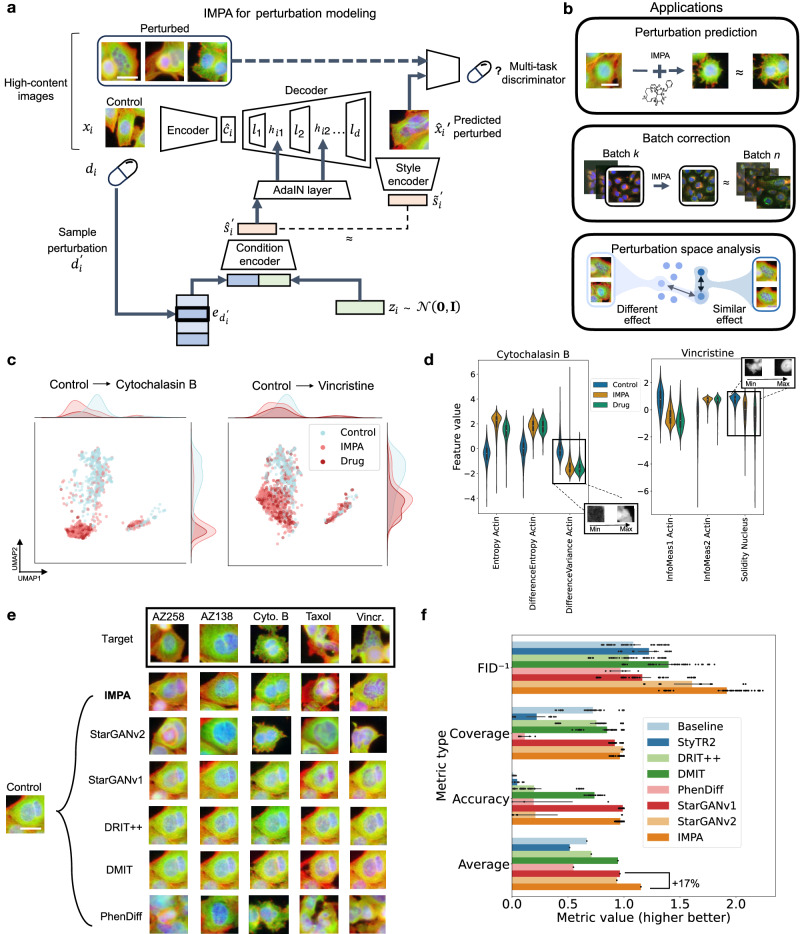
IMPA enables perturbation effect prediction via style transfer. **a** Perturbation prediction with IMPA. A control cell image *x*_*i*_ is encoded into a content representation while a dense embedding of the target perturbation is collected and concatenated with a random vector. A lower dimensional projection of the concatenation constitutes the style space which conditions every layer of the decoder via the AdaIN method. With *h*_*ij*_ we indicate the output of the *j*^*th*^ decoder layer on the image *i*. The transformed output leads a discriminator net to predict that the decoded image is a real example of the target perturbation. Moreover, a style encoder is trained to replicate the style vector from the transformed image. The scale bar is 20 μm. **b** Examples of use cases of the IMPA model: Prediction of morphological effects derived from applying a perturbation to cell images, correcting for technical variations by transporting images to a single experimental batch, learning a style space for perturbation where proximal perturbations are responsible for triggering a similar effect. The scale bar is 20 μm. **c** 2D UMAP plots of 356 CellProfiler features before and after transformation with IMPA for Vincristine and Cytochalasin B. Data points represent individual control, transformed control and real perturbation images in the test set of a five-drug subset of BBBC021 (*N* = 20,313). **d** Violin plots showing the distribution of discriminative CellProfiler features between controls (*N* = 520), IMPA’s predictions (*N* = 520), and original perturbation images for Vincristine and Cytochalasin B (*N* = 173 for Cytochalasin B and *N* = 354 for Vincristine). The boxes within the violin plots show the median, top and bottom quartiles of the feature distributions, while the whiskers mark the 95% quantiles. Real perturbed and control images are drawn from the test set. Source data are provided as Source data files. **e** Visual comparison of IMPA with existing models on the style transfer task. The scale bar is 20 μm. **f** Evaluation metrics comparing generated images with real perturbed images, averaged across different drugs on the BBBC021 dataset. Data are presented as mean values ± 95% confidence intervals. Source data are provided as Source data files.

We model the phenotypic responses to perturbations or technical effects in high-content imaging screens by decomposing the representation of each image into a perturbation or batch space (i.e., style) and a representation of the cell (i.e., content). This approach is based on style transfer^27^, an active area of research in deep learning and computer vision. Style transfer involves modifying the characteristics (e.g., the art style) of an image to another while preserving the original content of the image. Following this approach, we developed IMPA, a conditional GAN^19^ that generates the counterfactual image response to a desired chemical or genetic perturbation or transforms a microscopy image collected from an experimental source into another to remove batch effects (see Fig. 1a, b or Supplementary Fig. 1a, b). IMPA is built upon the architecture proposed in StarGANv2^30^. However, we applied modifications to the conditioning mechanism in a way that supports the usage of biologically informed perturbation embeddings and improves the scaling properties of the model (see Supplementary Fig. 2c, d for scaling plots and the Architecture section in the methods for details on the model). In the perturbation setting, this modeling aspect provides flexibility in the choice and representation of treatments, allowing for diverse examples such as incorporating molecular structures for drug screening, co-expression-based gene representations or DNA embeddings.

Given a dataset ${\left\{{x}_{i}\right\}}_{i=1}^{N}$ of $N$ high-content images of cells and the associated perturbation or batch index ${\left\{{d}_{i}\right\}}_{i=1}^{N}$, IMPA decomposes the latent representation of a cell ${x}_{i}$ into a content ${\hat{c}}_{i}$ and a style ${\hat{{s}}}_{i}$ component. The content ${\hat{{c}}}_{i}$ is inferred through a convolutional encoder that projects the input image onto a content space (see Tables 1 and 2 for additional information on the architecture of the encoder). To learn a representation for the style, we devise a condition and a style encoder (see Fig. 1a or Supplementary Fig. 1a, b) interacting together to learn an embedding for each perturbation or batch.

**Table 1:** The list of layers composing the residual architecture

Branch	Layer encoder	Layer decoder
Residual	IN	AdaIN
	LeakyReLU, *a* = 0.2	LeakyReLU, *a* = 0.2
	3 × 3 2D Conv.	2x NN-upscaling
	2 × 2 Avg. Pooling	3 × 3 2D Conv
	IN	AdaIN
	LeakyReLU, *a* = 0.2	LeakyReLU, *a* = 0.2
	3 × 3 2D Conv.	3 × 3 2D Conv.
Skip connection	1 × 1 2D Conv.	2 x NN-upscaling
	2 × 2 Avg. Pooling	1 × 1 2D Conv.

The layers are placed in the same order as they are found in the model. Encoder and decoder differ by the normalization method, the ordering and the dimensionality-altering layer. The encoder uses instance normalization and downsamples spatially via average pooling. The decoder is conditioned on the style through AdaIN and upsamples its input through the Nearest Neighbor (NN) interpolation.

**Table 2:** The list of layers constituting the generator network

Module	Layer	Dimension
Encoder	3 × 3 2D Conv.	64 × 96 × 96
	Down. ResBlock	128 × 48 × 48
	Down. ResBlock	256 × 24 × 24
	Down. ResBlock	512 × 12 × 12
	ResBlock	512 × 12 × 12
	ResBlock	512 × 12 × 12
Decoder	ResBlock with AdaIN	512 × 12 × 12
	ResBlock with AdaIN	512 × 12 × 12
	Up. ResBlock with AdaIN	256 × 24 × 24
	Up. ResBlock with AdaIN	128 × 48 × 48
	Up. ResBlock with AdaIN	64 × 96 × 96
	IN	64 × 96 × 96
	LeakyReLU	64 × 96 × 96
	1 × 1 2D Conv.	3 × 96 × 96

The entries Down. and Up. ResBlock derive from the modular structure described in Table 1, with, respectively, average pooling (encoder) or NN interpolation (decoder) layers. Their dimension-preserving counterpart is indicated as ResBlock. The Dimension column refers to the dimensionality of the output of each layer. In the table, we assume input spatial dimension of 96, but the same architecture works for larger images as well.

The condition encoder receives a concatenation between a noise vector ${z}_{i}$ and an embedding ${e}_{{d}_{i}^{\prime} }$ representing a perturbation or batch encoding ${d}_{i}^{{\prime} }$ different from the original ${d}_{i}$ and transforms said concatenation into a lower-dimensional style vector ${\hat{s}}_{i}^{{\prime} }$. The perturbation embedding choice is flexible. We employ Morgan Fingerprint descriptors^31^ for representing drugs and learnable embeddings to encode experimental batches. For genetic perturbations, we use a combination of target gene embeddings via Gene2Vec^32^ and DNA embeddings of CRISPR guides or Open Reading Frames (ORF) via HyenaDNA^33^ as the input of the condition encoder. The probabilistic nature of the perturbation style allows us to learn a full range of possible responses to each treatment in the training dataset when using IMPA as a treatment prediction tool. Since the style space is used to transform cells into their perturbed counterpart, proximity in the style space can be interpreted as similarity in the perturbation effect, providing an avenue to identify treatment clusters based on morphological activity (see Fig. 1b).

Using a convolutional decoder network, we perform conditional generation via Adaptive Instance Normalization (AdaIN)^34^ (see Table 1 and Table 2 for additional information on the architecture of the decoder). AdaIN manipulates the content encoding to match the input condition by scaling and shifting feature maps in the convolutional decoder based on the conditioning style. During training, IMPA combines random style embeddings and the content using AdaIN in each layer of the decoder (see Fig. 1a and the Architecture section in the methods for details). The generated image ${\hat{x}}_{i}^{{\prime} }$ is fed to a convolutional style encoder, which outputs a prediction ${\widetilde{s}}_{i}^{{\prime} }$ and is trained to approximate the output of the condition encoder ${\hat{s}}_{i}^{{\prime} }$ (see Table 3 for additional information on the architecture of the style encoder). Effectively, the style encoder links image features to a perturbation or batch-specific style. Such an approach enforces alignment between features extracted from the generated images and the learned style embedding, making them consistent.

**Table 3:** The discriminator and style encoder architectures implemented by IMPA

Module	Layer	Dimension discr.	Dimension style enc.
Discriminator	3 × 3 2D Conv.	64 × 96 × 96	64 × 96 × 96
	Down. ResBlock	128 × 48 × 48	128 × 48 × 48
	Down. ResBlock	256 × 24 × 24	256 × 24 × 24
	Down. ResBlock	512 × 12 × 12	512 × 12 × 12
	Down. ResBlock	512 × 6 × 6	512 × 6 × 6
	Down. ResBlock	512 × 3 × 3	512 × 3 × 3
	3 × 3 2D Conv.	*p* × 1 × 1	*s* × 1 × 1
	Reshape	*p*	*s*
	Sigmoid	*p*	-

With *p*, we indicate the number of perturbation categories, whereas *s* is the dimensionality of the style space. In the table we assume input spatial dimension of 96, but the same architecture works for larger images as well. The Dimension column refers to the dimensionality of the output of each layer.

To ensure that predictions correctly match the desired perturbation or batch style, we train a single discriminator with one classification head for each condition (batch or perturbation) to discriminate between real images of cells from such a condition versus generated images (see Table 3 for additional information on the architecture of the discriminator). The decoder network must synthesize accurate images of the effect of the target ${d}_{i}^{{\prime} }$ to deceive a multi-task discriminator into classifying it as a true image from the target style (batch or perturbation). Conversely, the discriminator is trained on real data to recognize the difference between a true and a generated treatment image. The multi-task discriminator does not attempt to classify images between different conditions but to predict whether an image is true or generated according to a perturbation class. This approach is more suitable for the treatment prediction setting when the phenotypic responses to different perturbations are similar and, as such, hard to classify. Finally, a cycle-consistency loss^35^ is employed to encourage the model to learn reversible mappings (see Supplementary Fig. 1b), hence, transferring transformed images back to the style of the input condition.

### IMPA accurately predicts morphological changes after drug perturbations

We evaluated IMPA on predicting morphological changes after drug perturbation using the BBBC021 dataset (see Datasets)^36^. BBBC021 comprises images of p53-wildtype breast cancer model cells (MCF-7) perturbed with 112 compounds and imaged across three channels: nucleus, β-tubulin and actin. Initially, we employed a widely used reduced version of the dataset from Ljosa et al.^12^ comprising compounds with expected phenotypic effects at the tested concentrations (see Supplementary Table 1 for a list of compounds and concentrations). We pre-processed the dataset from whole slides to images cropped around single cells. For a more straightforward assessment of generated images, we first trained our model on a subset of five drugs (AZ138, AZ258, Cytochalasin B, Taxol, Vincristine) with visible effects on the morphology compared to controls according to Ljosa et al.^12^ (20k images). For this scenario, a perturbation embedding for each drug was extracted as Morgan Fingerprints^31^ using the RDKit software package. Morgan fingerprints are a type of binary molecular fingerprinting method used in cheminformatics to encode structural information of molecules for similarity searching and machine learning applications^37^.

To assess the impact of IMPA on important features characterizing perturbations, we employed the CellProfiler software^38^ to compute a series of 356 features, including shape, texture, area, and intensity distribution of cell images before and after transformation by IMPA. Our objective was to investigate how IMPA affects these features and their alignment with actual perturbed images compared to control cells. Visual inspection of UMAP dimensionality reduction plots from the features extracted from real and transformed images revealed that CellProfiler features of generated images aligned better with real perturbed images than controls, corroborating our observation that the model alters biological features coherently with the expected perturbation outcome (see Fig. 1c for Vincristine and Cytochalasin B and Supplementary Fig. 3a for all five drugs). To subset relevant features for each perturbation, we estimated feature importance by training a Random Forest classifier using CellProfiler features to discriminate control cells from perturbed cells in separate binary classification settings. The distribution of important morphological features in transformed images showed a similar shift to actual perturbed images when compared to controls (see Fig. 1d and Supplementary Fig. 3b). For example, we can see an increase in actin entropy in Cytochalasin B (actin disruptor) or a loss of nuclear solidity in Vincristine (tubulin destabilizer causing cell death) in both real and generated treated cells.

We compared IMPA with six existing models performing the style transfer task (see the Baselines section in the methods). Four of such models (DRIT + + ^39^, DMIT^40^, StarGANv1^41^ and StarGANv2^30^) are also GAN models. In the group of GAN-based models, only IMPA and StarGANv2 incorporate a multi-task discriminator to guide the generation process, while the other methods adopt a multi-class convolutional classifier. Moreover, we included two additional baselines: PhenDiff^22^ and StyTR2^42^. The former is a diffusion-based model specifically developed for drug effect prediction. The latter is a standard transformer model for style transfer. Qualitatively, our visual analysis (Fig. 1e) revealed that DRIT + + did not produce significant deviation from the source images, while StarGANv1 and DMIT successfully altered intensity distributions but tended to preserve control morphology. Meanwhile, some of PhenDiff’s outputs lack a clear cell shape. The visual results produced by StarGANv2 are the most convincing among the baselines, although the model struggles to capture phenotypic patterns such as the loss of nuclear integrity caused by Vincristine. We did not include visual results from StyTR2 since we found that transformations did not depart significantly from the source control image. In contrast with most of the compared approaches, the results obtained from IMPA visually approximate the target phenotype for all tested compounds.

To quantitatively compare these models, we employed two commonly used evaluation metrics for generative models: Fréchet Inception Distance (FID)^43^ and Coverage^44^, which assess the similarity between the distribution of generated images and the target real image distribution. In addition, we measured an accuracy metric that evaluates how often a pre-trained classifier correctly labels generated images with the MoA known for the target drug (see the Evaluation and metrics section in the methods for a detailed description of the evaluation process). To establish a lower-bound performance, we included a baseline comparison between control cells and real perturbed images, simulating a model incapable of style transfer. Our findings demonstrated that IMPA outperformed the other style transfer models in FID and achieved an overall 17% performance increase compared to the second-best method. On the Coverage metric, IMPA is marginally overcome by StarGANv2, probably due to its more expressive style encoding mechanism. However, StarGANv2 also produces inconsistent perturbation images in terms of MoA features, as demonstrated by a low classification accuracy. Notably, on such a metric IMPA performed comparably well to StarGANv1, exhibiting only a 2% decrease in classification accuracy, while significantly outperforming all other models. It is worth mentioning that IMPA gains in both scalability and overall performance compared to StarGANv2 (see Supplementary Fig. 2c, d), as the conditioning mechanism implemented by the latter is based on using a separate neural network for encoding conditions and cannot deal with scenarios involving large perturbation pools. Conversely, IMPA exploits perturbation embeddings and encodes them to a style space with a shared neural network, reducing its number of parameters significantly. Finally, as a GAN-based model, IMPA is faster at inference than PhenDiff (see Supplementary Table 2) since diffusion models require simulating a stochastic differential equation for generation.

### IMPA captures population changes and generalizes to unseen perturbations

**Fig. 2:**
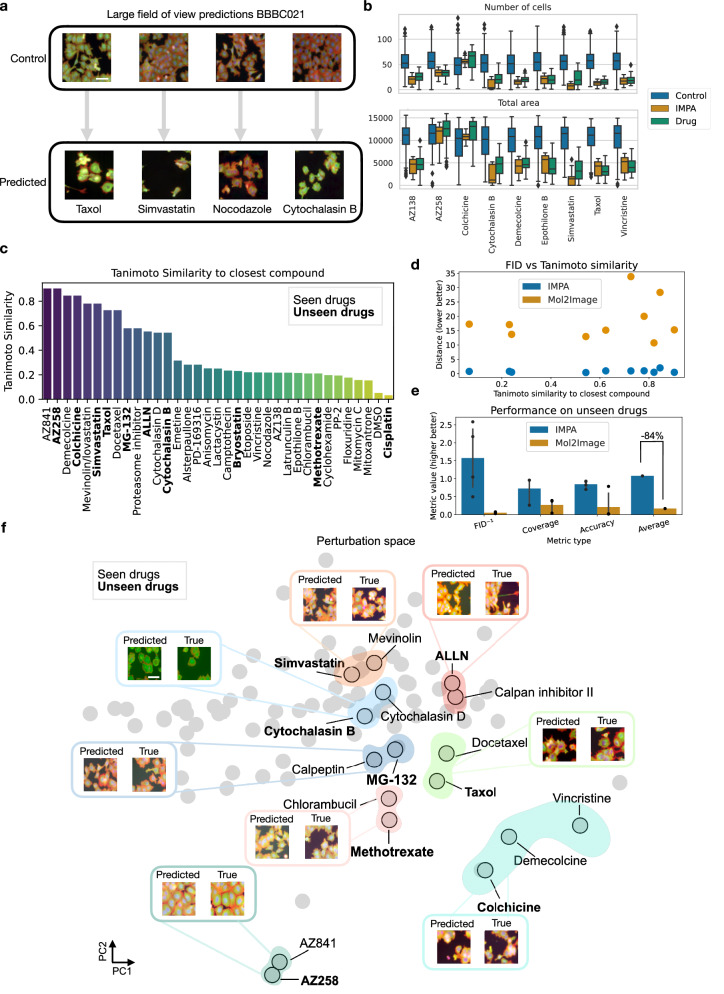
IMPA predicts population response to perturbations and unseen drug effects on the whole BBBC021 dataset (*N* = 118,799). **a** Large field of view prediction of the perturbation response to Taxol, Simvastatin, Nocodazole and Cytochalasin B performed by IMPA. The compounds have distinguishable effects both on morphology and cell density. The scale bar is 30 μm. **b** The distribution of the number of cells and total cell area before and after IMPA’s transformation computed for 10 drugs and compared with real perturbed cells. The boxplots show the median, top and bottom quartiles of the considered features across settings. The whiskers in the boxplots mark the 95% quantiles. Source data are provided as Source data files. **c** The Tanimoto similarity of each compound and its closest perturbation in BBBC021. Compounds highlighted in bold were held out from training via scaffold-based splitting. For brevity, only 35 drugs out of the 99 in the dataset are shown in the plot. Source data are provided as Source data files. **d** Model performance on held-out compounds in terms of FID as a function of their Tanimoto similarity to the closest training drug. Source data are provided as Source data files. **e** Comparison between IMPA and Mol2Image on the unseen drug's effect prediction tasks. For all measurements, the higher the value, the better the generated output approximates the expected phenotype. Metrics are averaged across the 10 unseen drugs. MoA prediction accuracy is evaluated only on the 4 visually annotated drugs (AZ258, Colchicine, Taxol and Cytochalasin B) in the unseen group. Data are presented as mean value ± 95% confidence intervals. Source data are provided as Source data files. **f** 2D PC plot of the perturbation space learned by the style encoder. Perturbations highlighted in bold are part of the set of held-out compounds. Groups are highlighted as drugs triggering a similar phenotypic effect in the original dataset. Examples of predictions by IMPA on the unseen perturbations in the groups are provided together with images displaying the real phenotype. The scale bar is 30 μm.

Moving beyond single-cell changes, we also tested IMPA on the task of capturing cell population behaviors together with single-cell morphological responses. We applied IMPA to larger fields of view derived from the BBBC021 dataset. This aspect is crucial for the applicability of the model, as it shows that IMPA not only captures fine-grained morphological variations but is also able to predict shifts in cellular density. In the examples provided in Fig. 2a, IMPA successfully captures the cellular depletion together with the morphological effects triggered by cytotoxic compounds such as Taxol, Simvastatin, Nocodazole and Cytochalasin B (see Supplementary Fig. 2b for more examples). This observation is quantitatively corroborated by the change in the number of cells and total covered area metrics (see Fig. 2b) induced by our model, where we see that the IMPA-mediated transformations approximate the expected measurements in the real drug plates for most of the considered perturbations. Moreover, IMPA can predict the absence of effect when using DMSO as a perturbation key (see Supplementary Fig. 2e for visual examples).

A long-standing challenge for drug screening is selecting a chemical library to cover different areas of the chemical space^45–47^ corresponding to diverse MoAs. However, fully exploring all possible compounds is impractical due to experimental costs and logistics^4,48^. Therefore, in-silico methods are needed to predict the response to unmeasured compounds. IMPA’s architecture allows for tackling this challenge by simply using chemical representations for unseen drugs at test time. In this section, we extend our analysis to the entire BBBC021 dataset, consisting of a cohort of 99 drugs with known structures (118k images). A key characteristic for predicting unseen perturbations is the model’s ability to generate intermediate phenotypes through interpolations in the perturbation data’s style space. This aspect is crucial when computing unseen drug predictions, as the model requires a smooth style space to be able to associate an unseen perturbation style to a feasible phenotype. Supplementary Fig. 5b illustrates linear interpolations from control to perturbation embeddings, decoding intermediate phenotypes for Cytochalasin B (actin disruptor) and Vincristine (tubulin destabilizer). These interpolations visually demonstrate a smooth transition in the phenotypic landscape, reflected by gradual changes in specific morphological features like increased actin contrast and decreased nuclear area due to apoptosis.

Equipped with the evidence of a smooth style space, we proceeded to demonstrate that IMPA can predict the effect of unseen perturbations as interpolations of the chemical representation space. For the sake of the experiment, we left out ten compounds as an unseen test set. Splitting was performed via the scaffold-based approach^49^, which splits compounds based on structural components in the molecules to ensure train and test diversity. The held-out set was obtained from a subset of the dataset with annotated MoA to aid evaluation. Reasonably, the ten compounds left out of the training process exhibited different levels of Tanimoto similarity to training drugs (Fig. 2c and Supplementary Table 3). Tanimoto similarity is a metric that measures the similarity between two molecular fingerprints, with a range from 0 (no similarity) to 1 (identical molecules). In this study, we also directly compared our approach, IMPA, to Mol2Image^21^, a flow-based generative model that generates images of perturbed cells conditioned on learned graph-based drug representations. The core difference is that Mol2Image generates images of perturbed cells conditionally from Gaussian noise, while IMPA performs style transfer on existing images conditioned on prior perturbation embeddings. Thus, our approach has the advantage of enabling the study of morphological shifts between controls and perturbed conditions, providing a deeper understanding of the impact of drug-induced changes on cellular phenotypes.

In Fig. 2d, we showed that IMPA consistently achieved a lower FID score compared to Mol2Image on all the held-out compounds. Moreover, the performance of our model appeared stable across test drugs, irrespective of their Tanimoto similarity to the closest training compounds. This result suggests that the prediction quality does not significantly degrade with chemical species different from training compounds. In Fig. 2e we report average performance scores for both models when predicting the morphology of held-out compounds. The accuracy metric was evaluated only on AZ258, Colchicine, Taxol and Cytochalasin B since they induce the visually annotated MoAs described in Ljosa et al.^12^ distinguishable by a classifier. The rest of the metrics were evaluated on the whole held-out set. Overall, Mol2Image’s average performance across the evaluation metrics showed an 84% decrease compared to IMPA’s performance. This gap was particularly relevant in terms of MoA classification accuracy: While Mol2Image rarely produces realistic drug response outputs, IMPA’s predictions of morphological responses are often labeled correctly, making it a more reliable model for untested treatment response generation. Finally, IMPA scales better than Mol2Image as a function of the number of perturbations considered (see Supplementary Fig. 2d).

Finally, we evaluated if the perturbation space predicted by IMPA contains a meaningful biological structure. In Fig. 2f we reported the 2-dimensional Principal Component (PC) representation of the style space learnt by IMPA for seen and unseen drugs. Notably, proximity in the chemical space corresponds to drugs causing similar visual phenotypes (see Supplementary Fig. 6 for examples of images of cells treated with drugs encoded close to each other). As expected, couples of seen and unseen compounds with very similar functions and structures tend to be embedded proximally to each other. This is valid for Cytochalasins B and D (Tanimoto similarity of 0.54), AZ258 and AZ841 (Tanimoto similarity of 0.90), Simvastatin and Mevinolin (Tanimoto similarity of 0.78) and Taxol and Docetaxel (Tanimoto similarity of 0.73). However, IMPA also captured more interesting patterns. For example, IMPA represents Methotrexate and Chlorambucil close to each other although the compounds only share a Tanimoto similarity of 0.21. This reflects their functional similarity as agents interfering with DNA replication^50,51^ and their relatedness is visually validated in Supplementary Fig. 6. Likewise, IMPA predicts a similar response of tubulin destabilization using Vincristine, Colchicine and Demecolcine. Such a result confirms the capability of our model to relate functionally similar drugs despite their chemical distance, as Vincristine has a Tanimoto similarity of 0.19 and 0.17 to Colchicine and Demecolcine, respectively. Furthermore, IMPA produces close representations and morphological responses for ALLN (a Calpain I inhibitor) and Calpain inhibitor II, reflecting their functional affinity. Finally, also Calpeptin and MG-132 are encoded close to each other and trigger a similar response (Tanimoto similarity of 0.62). Functionally, they are both Calpain inhibitors^52^. Aside from the chemical space structure, IMPA produces response predictions close to the original unseen perturbation images (see Fig. 2f), further confirming the synthetic perturbation capabilities of our model.

In summary, IMPA learns a phenotypically meaningful perturbation space and uses it to induce a morphological effect on control cells. One can interpolate such a space to derive predictions of the effects of unmeasured compounds lying between observed chemical species.

### IMPA corrects technical variation in multi-batch high-content imaging screens

One of the major challenges in modern large perturbation screenings is the arising of technical effects as potential confounders for the treatment signal. Testing hundreds of perturbations in parallel often requires collecting microscopy readouts from different sources, which can be both experimental batches and sampling locations^11^. When performing simple perturbation analyses or predictions it is important to account for experimental effects to avoid relating spurious technical variation to perturbation outcomes. Recent studies have explored the efficacy of batch correction algorithms applied to featurized versions of cellular images^53^. While such methods output a latent representation of morphological features with a lower technical effect, they do not offer access to batch-corrected images. Exploiting the analogy with perturbation prediction, we explored the application of IMPA to the task of technical effect removal via style transfer.

**Fig. 3:**
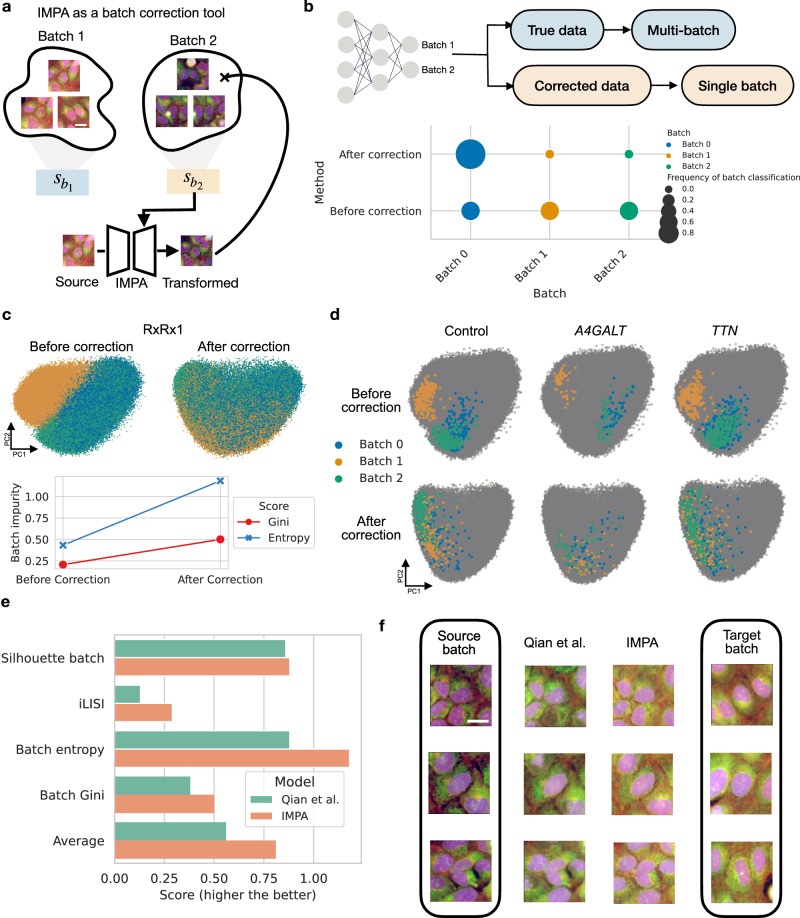
IMPA corrects technical batch effects via style transfer on RxRx1 (*N* = 170,942). **a** Given a dataset of images collected from multiple batches, a style embedding is learnt for each batch and used to transport all images into the same batch. The scale bar is 20 μm. **b** A classifier is trained to distinguish cells from different batches. Before correction, cells should be assigned to their original batch. After correction, the classifier is deceived into labeling all the cells with a single batch. The dot plot represents the fraction of cells assigned to each batch by the classifier before and after correction by transforming all images to batch 0. Source data are provided as Source data files. The Scale bar is 20 μm. **c** Top - PCA plots before and after correction colored by batch labels. The features are extracted with a pre-trained Cell Painting Vision Transformer (ViT). Bottom - mean batch impurity scores are measured as entropy and Gini index computed for each cluster of images. A higher value of batch impurity suggests a better mixing of batch labels within a cluster. Clusters are derived using the Leiden algorithm. Source data are provided as Source data files. **d** Highlighted cell images before and after correction by IMPA colored by batch for controls and treated with siRNAs targeting *A4GALT* and *TTN* genes. **e** Metrics comparing batch correction results between IMPA and a competing model evaluated on the ViT features extracted from corrected images. Source data are provided as Source data files. **f** Visual examples of transformations from batch 1 to batch 0 across models. The scale bar is 20 μm.

Figure 3a provides a visual depiction of the modified objective. Images from different experimental sources are used to train a style transfer model able to transport images from batch to batch (see Supplementary Fig. 7a, b). Similar to the perturbation use case, to each batch we associate a style embedding used by the model to transform images across sources. However, differently from the perturbation case, the style embedding is here learnable and not based on a prior knowledge encoding. A batch-corrected version of the dataset can be obtained by transporting all images to a single template source upon training. Given IMPA’s fast inference times, one can use the model to perform correction while training perturbation prediction, without storing a batch-corrected version of the dataset in memory.

We showcase the performance of IMPA on the batch correction task using the RxRx1 dataset^54^, where we specifically select a subset of U2OS osteosarcoma cells perturbed with more than a thousand siRNAs in three distinct experimental batches (170k images). As a first evaluation step, we demonstrate that source-corrected images generated by IMPA are realistically transported to a single batch to remove technical confounding. To assess this aspect, in Fig. 3b we train a convolutional classifier predicting the batch starting from perturbation images. As the batch effect is strong in the dataset, the classifier achieves almost 100% classification performance, making the ability to deceive it even more meaningful. Before correction (bottom row of the dot plot), the classifier assigns the images to separate batches uniformly. Oppositely, after correction (top row of the dot plot) the classifier predicts that most images originate from batch 0, the one chosen as a target for the style transfer process. We further illustrate the application of IMPA to the batch correction task by featurizing the cell images with a pre-trained Cell Painting Vision Transformer (ViT)^55^ and examining how the embedding changes before and after batch correction. Figure 3c displays the results in 2 dimensions using a PC projection of the featurized data. Visually, uncorrected data display remarkable heterogeneity between sources, with batch 1 being visibly different from the rest of the batches. Correcting for such phenomenon with IMPA induces mixing between images from different sources. To quantify such a mixing, we cluster the featurized images before and after correction using the Leiden algorithm^56^ and compute how much each cluster is impure with respect to the batch label in terms of entropy and Gini index (see the bottom panel of Fig. 3c). An increase in mean impurity after correction quantitatively confirms our visual analysis and illustrates a successful integration of images from batch 1 into the counterpart cluster formed by batch 0 and 2.

Applying batch correction to the dataset is a fundamental aspect of unveiling a morphological consensus between cells to which the same perturbation has been applied. In Fig. 3d and Supplementary Fig. 7d, we demonstrate how the absence of batch effect is essential for cellular responses to the same perturbation to correctly aggregate with each other. Indeed, before applying IMPA-mediated batch correction, untreated cells and genetically perturbed cells on genes *A4GALT* and *TTN* cluster based on experimental sources. This represents an issue for downstream applications, where the perturbation effects are usually unveiled by comparison with controls, which are expected to occupy a distinct portion of the morphological space.

To put IMPA’s performance into context, we compared its batch effect correction performance with that obtained using the model introduced by Qian et al.^24^. More in detail, we extracted morphological features from the corrected images from both models using the aforementioned ViT architecture. In Fig. 3e, we quantify the batch correction using two metrics from the scIB package (silhouette batch, iLISI)^57^, which evaluate batch mixing considering data neighborhood compositions. Moreover, we consider batch label impurity through the Gini index and entropy metrics computed over Leiden clusters of the corrected data as additional measures of correct batch equalization. On all metrics, IMPA surpasses the competing method, producing an average improvement of 45%. In Fig. 3f, we visually validate improved batch effect performance by IMPA, showing that our model’s results better approximate the target batch images.

Finally, in Supplementary Fig. 8a-d we demonstrate that IMPA successfully corrects images from unseen batches. More specifically, we leave out batch 1 of U2OS cells and train the model on the remaining technical batches (Supplementary Fig. 8a). Images from batch 1 are then corrected by transforming them to batch 0. We evaluate the integration of unseen sources of variation showing that our model successfully mixes held-out batch features with those from existing data (Supplementary Fig. 8b). Moreover, a classifier trained to recognize batches assigns the batch 0 label (target of the transformation) to 99% of unseen batch cells equalized via IMPA (Supplementary Fig. 8d). Such a result is relevant for big microscopy datasets, as it shows that our model generalizes to incoming experimental batches without re-training.

### IMPA predicts diverse perturbation types via learning a shared perturbation space

**Fig. 4:**
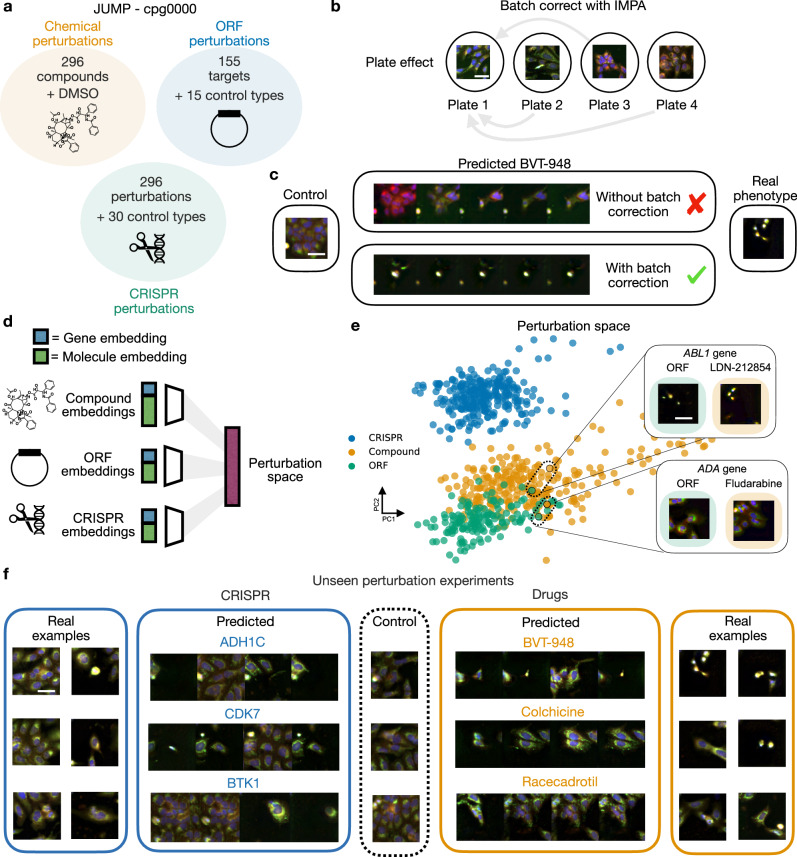
IMPA predicts the effect of multiple perturbation types on cpg0000 (*N* = 435,160). **a** An overview of the types and number of perturbations in the JUMP-cpg0000 dataset. We consider 435,160 images of treated U2OS cells. **b** The first step before predicting perturbation responses is to use IMPA to remove the plate effect. Scale bar is 30 μm. **c** Illustration of the importance of removing plate effect with IMPA for perturbation prediction. Above, are the predictions of the effect of BVT-948 without performing plate correction. Below, are the results after plate correction. On the right, is an example of an expected phenotype. Scale bar is 30 μm. **d** Depiction of the different perturbation embeddings as input to IMPA’s perturbation encoder to learn a shared perturbation space. **e** PCA plot of the perturbation embedding computed by IMPA colored by perturbation type. Highlighted are two different couples of drugs and ORF perturbations proximal in the perturbation space that target the same genes. Scale bar is 30 μm. **f** Morphological predictions of three unseen drugs and CRISPR perturbations computed by IMPA. At the centre, the control image is used as an input for the prediction. For each perturbation, four generated outputs are computed by drawing four random codes during inference. On the sides are two examples of real cells from the chosen perturbation. Scale bar is 30 μm.

Modern phenotypic screenings involve multiple perturbation types, such as small molecules, CRISPR knockouts and Open Reading Frame (ORF) overexpression assays to obtain a holistic view of drugs and their potential targets. The recent JUMP-cpg0000 dataset^58^ is an example of such a combination of assays, where CRISPR knockouts, drug perturbations and ORF overexpression assays are screened across multiple cell lines using the Cell Painting protocol. To demonstrate the flexibility of IMPA at learning perturbation effects across multiple perturbation types, we introduce a shared perturbation space across different treatment modalities. Here, we use the JUMP-cpg0000 dataset and focus on the U2OS cell line, considering 296 drugs, 155 ORF overexpression perturbations and 296 CRISPR guides (see Fig. 4a). Two different CRISPR guides, two chemical perturbations and one ORF reagent were designed for each targeted gene in the original study. After pre-processing via illumination correction and splitting the images into patches, we yield 435,160 cell images as a dataset for the application of IMPA.

As we previously demonstrated, the presence of batch effects can lead to a biased representation dominated by technical variations. To account for this, we perform plate correction with IMPA before running perturbation prediction (see Fig. 4b). The importance of performing batch correction before predictions with IMPA is displayed in Fig. 4c and Supplementary Fig. 9. As an example, we show the qualitative performance of IMPA on the Protein Tyrosine Phosphatase (PTP) inhibitor BVT-948, a drug causing a strong cell phenotype. Predicting perturbations on a confounded dataset (upper row) leads the model to fit technical effects and miss the target phenotype, which is instead perfectly captured on a previously corrected dataset.

To perform predictions on unseen genetic perturbations as well as on drugs, we designed a multi-type perturbation space using embeddings for each perturbation type. Specifically, our goal is to define embeddings that retain perturbation-specific information (such as the physiochemical properties of a drug or the seed sequence of a CRISPR guide) while learning the phenotypic similarity between different perturbation types guided by Cell Painting data. As a shared type of information, we use the Gene2Vec embedding of the gene targeted by both genetic and chemical perturbations (see Fig. 4d). The Gene2Vec embedding is concatenated with a modality-specific embedding. We use Morgan Fingerprints for drugs and HyenaDNA to embed CRISPR guides and the ORF 5’ flanking sequence in the overexpression assays. Each modality has its condition encoder that maps from the representation space of a treatment type to the perturbation embedding used for style transfer.

We observed an overlap between drugs and ORF perturbations, while CRISPR perturbations cluster separately due to differences in their nature and level of efficacy, as described in Chandrasekaran et al.^58^ (see Fig. 4e and Supplementary Fig. 10c). For example, the compound LDN-212854 and the ORF reagent targeting the Adenosine Deaminase (*ADA*) are close to each other. Association in the effect of *ADA* with Adenosine inhibitors like LDN-212854 was also observed by Chandrasekaran et al.^58^. Similarly, the representations of the ORF targeting *ABL1* and Fludarabine, which also targets the same gene, are proximal. We extended this analysis to focus on drugs with known modes of action and observed that many drugs with similar mechanisms are closely embedded, further highlighting IMPA’s ability to group perturbations with similar effects (see Supplementary Fig. 10a). This also holds for different CRISPR perturbations targeting the same gene (see Supplementary Fig. 10b). Overall, the perturbation space learned by IMPA can serve as an analysis toolbox to compare various types of perturbations, as well as for identifying new drug targets.

Learning a shared perturbation space allows for morphological predictions on both unseen drugs and genetic perturbations. We focus on unseen CRISPR guides and compounds, which show a stronger signal than ORF perturbations. In Fig. 4f, we provide qualitative insight into unseen perturbation predictions using three examples of CRISPR and compound perturbations. Active perturbations were selected by visual inspection and literature search. For both perturbation types, IMPA correctly captures the main morphological behavior of treated cells. While for drugs this effect is more uniform, multiple generations of CRISPR perturbations yield variable effect sizes. This is in line with the observed data since the emergence of morphological effects of CRISPR perturbations is less consistent than in chemical perturbations (see Fig. 4f and Supplementary Fig. 10c for more examples). In other words, the generative model properties of IMPA allow for it to learn the response to unseen perturbations both when they have a consistent (drugs) and variable (CRISPR) effect.

## Discussion

Synthetic prediction of morphological responses to perturbation in high-content screenings is important to guide experimental design towards promising treatments. To this end, we implemented IMPA, a conditional generative adversarial network that morphs images of untreated cells into what they would look like under the effect of a queried perturbation. IMPA derives treatment-specific encodings and uses them as a style to overlay the phenotypic effect due to perturbations onto control cell images. More specifically, we interpreted the problem of predicting perturbations on cells as disentangling content from style information on input images. Moreover, when replacing perturbations with sources of technical variation for the style transfer process, IMPA can be used as a batch correction tool by transporting all images from separate acquisition sources into the same batch.

We demonstrated the use of IMPA on different types of perturbation data. In drug screenings, we derived the style encoding to condition image translation from dense molecular embeddings extractable from any compound with a known structure and showed that IMPA successfully approximates target perturbations qualitatively and quantitatively, outperforming six other image-to-image translation models in the task. Moreover, IMPA’s ability to use structural information from drugs to condition perturbation style transfer allows the model to predict the phenotypic effect of unseen treatments, interpolating the chemical space derived from training compounds. We also showed that IMPA can combine chemical treatments with other types of perturbations and learn a joint perturbation space encompassing drug, CRISPR and overexpression assays. In this setting, perturbation embeddings consist of combinations of chemical representations, co-expression-based target gene encodings and DNA sequence embeddings. Finally, we showed the effectiveness of IMPA as a batch-effect removal tool, illustrating how corrected results are an important asset for unconfounded perturbation analysis.

Potential limitations of our model reside in its heavy dependence on the specification of the embeddings used to learn a perturbation style, where proximity between perturbations should be related to phenotypic impact. In other words, in settings where chemical proximity is not directly associated with a phenotypic response, IMPA may fail to generalize to unseen compounds. Moreover, IMPA is not able to extrapolate to unseen phenotypes, since it is trained to associate responses explored during training to the perturbation space. The model should rather be intended as a perturbation space interpolation approach to discover novel areas associated with phenotypes existing in the training set. The performance of IMPA is also influenced by the quality and amount of signal in the treated images, which are liable to noise and low perturbation penetrance. This aspect is an issue in modern phenotypic screenings, where the increased throughput is usually causative for a less clean signal. Moreover, it should be stressed that, while IMPA can predict unseen compounds and genetic perturbations, its predictive ability and performance for perturbations that are very different from those in the training set may decrease. Therefore, we encourage users to always validate their results both computationally and experimentally. The performance can be improved when trained on a diverse set of perturbations with different chemical and phenotypic characteristics, and the awareness of the model’s limitations is crucial to better guide rational experimental design.

 Future work will investigate the relationship between the chemical space and morphological changes more deeply. This will involve exploring how different regions of the chemical space are associated with specific morphological transformations. By understanding this relationship more deeply, we can enhance our ability to interpret and predict phenotypic responses to various drugs and perturbations. Furthermore, our effort can be extended to more sophisticated methods for condition encoding. For example, instead of using handcrafted descriptors, we might explore embedding drug treatments using modern deep-learning featurization paradigms pre-trained on thousands of compounds. So far, most of the proposed phenotypic screenings focus on either one or a small number of cell lines. Thus, generalizing to unseen model systems is complicated, especially in contexts where distinct cell lines respond differently to the same perturbation. Eventually, with the deployment of larger high-content screening studies profiling multiple cell lines at the same time, we hope that future resources will allow us to test IMPA on learning new cell line responses.

## Methods

### Model

#### Problem statement

Let ${\left\{({x}_{i},{d}_{i})\right\}}_{i=1}^{N}$ be an image dataset where ${x}_{i}\in {{\mathbb{R}}}^{C\times H\times W}$ represents the ${i}^{{th}}$ cell image and ${d}_{i}\in\{1,\ldots,p\}$ the index of the perturbation or the technical batch label associated with ${x}_{i}$, which we refer to as a condition. $N$ is the size of the dataset and $p$ is the total number of condition categories. Additionally, let the variable ${z}_{i}\sim N(0,I)$ be a random noise vector drawn from a $K-$ dimensional multivariate Gaussian and ${e}_{j}\in {{\mathbb{R}}}^{D}$, with $j\in\{1,\ldots,p\}$, be condition embeddings for each treatment. We assume that the data is derived from a generative process conditioned on a content *c* and a condition-specific random style vector $s\in {{\mathbb{R}}}^{S}$. Specifically, we model the style encoding as a function ${\hat{s}}_{i}=f({e}_{{d}_{i}},{z}_{i})$, where $f$ is a linear projection $f:{{\mathbb{R}}}^{D+K}\mapsto {{\mathbb{R}}}^{S}$. We infer the content ${\hat{{c}}}_{i}$ from the image ${x}_{i}$. The embeddings ${e}_{{d}_{i}}$ can be either fixed and obtained from a prior representation of the treatments or randomly initialized and trained.

Our goal is to approximate the unknown conditional probability distribution ${p}^{*}\left(x | c,s\right)$ through a parametrized generative model ${p}_{\theta }\left(x | c,s\right)$ that can be actively sampled. In this framework, the representations $c$ and $s$ respectively control the visual content of the generated sample and its condition-specific style. In the ideal case where our learned ${p}_{\theta }$ converges to ${p}^{*}$, any sample ${\hat{{x}}}_{i}$ from ${p}_{\theta }(\cdot |{\hat{{c}}}_{i},{\hat{s}}_{i})$ appears like real examples of cells treated with condition ${d}_{i}$ conserving visual features encoded by ${\hat{{{\rm{c}}}}}_{i}$

Under such a formulation, our model can perform the transformation of data point ${x}_{i}$, coming from the real ${d}_{i}^{{th}}$ condition modality and with content ${\hat{{c}}}_{i}$ to an inferred ${\hat{x}}_{i}^{{\prime} }$ image associated with condition ${d}_{i}^{{\prime} }$ different from ${d}_{i}$. This is achieved by sampling from ${p}_{\theta }\left(\cdot | {\hat{{c}}}_{i},{\hat{s}}_{i}^{{\prime} }\right)$, where ${\hat{s}}_{i}^{{\prime} }$ is the style derived from ${d}_{i}^{{\prime} }$. Specifically, ${\hat{x}}_{i}^{{\prime} }$ represents what the input ${x}_{i}$ would look like under a different condition type. In what follows, we describe the implementation and training process of IMage Perturbation Autoencoder (IMPA), a generative adversarial model performing phenotypic transformations on cell perturbation images and technical effect correction via style transfer.

#### Training

We develop our model as a conditional Generative Adversarial Network (cGAN) for image-to-image translation. The architecture and underlying working principles are inspired by StarGANv2^30^. The main backbone of the architecture is constituted by an image encoder $E$, a decoder $D$, a discriminator ${Dis}$, a style encoder ${E}_{{sty}}$ and a condition encoder $f$.

During training, the input tuple $({x}_{i},{d}_{i})$ is fed to the model. The image ${x}_{i}$ is used to infer the content ${\hat{c}}_{i}$ as follows:

1 $${\hat{c}}_{i}=E\left({x}_{i}\right)$$

where $E$ models the content space. To teach the model how to induce a distribution shift on the images to a different condition, we perform input transformation during training. In practice, we use different training strategies depending on the use case of IMPA:

- For perturbation prediction, we only learn to convert controls into perturbed cells. Therefore, we learn a one-to-many mapping, where ${x}_{i}$ in the input tuple $({x}_{i},{d}_{i})$ is a control cell that the model needs to convert to its treated version under a perturbation $d_i^{\prime}$ sampled from the existing perturbation pool (which can also include control as a condition).
- In the presence of multiple perturbation types (e.g., drug and genetic perturbations), each treatment type has its style encoder network, projecting perturbation embeddings into a shared perturbation space.
- For batch correction experiments, we instead train to transport each batch into any other via a many-to-many prediction model. More specifically, for each training pass, a condition ${d}_{i}^{{\prime} }$ is sampled from a discrete uniform distribution over indices $I-\{{d}_{i}\}$, with $I=\{1,\ldots,p\}$ and where *d*_*i*_ is the batch from which observation ${x}_{i}$ has been sampled.

In all cases, ${d}_{i}^{{\prime} }$ is used to select the embedding ${e}_{{d}_{i}^{\prime} }$ of the corresponding condition. IMPA is a probabilistic generative model. To induce a non-deterministic style transition, we concatenate ${e}_{{d}_{i}^{\prime} }$ with a random noise vector ${z}_{i}$ and encode such combination to a style vector via $f$ as:

2 $${\hat{s}}_{i}^{{\prime} }=f({e}_{{d}_{i}^{\prime} },{z}_{i})$$

The derived style is used to condition the generative model function approximated by the decoder $D$. In this regard, the style vector ${\hat{s}}_{i}^{{\prime} }$ is meant to induce the generator to decode ${\hat{{c}}}_{i}$ into an image ${\hat{x}}_{i}^{{\prime} }$ with characteristics typical of condition ${d}_{i}^{{\prime} }$. For brevity, we refer to the combination between $E$ and $D$ as generator and indicate it with $G$. Specifically, we refer to the succession of encoding and decoding passes as $G(x,s)=D(E(x),s)$.

To favor realism in the synthesized image, we couple the described autoencoder architecture with a multi-task discriminator ${Dis}$. Provided an input, the discriminator yields $p$ different probability values, one for each condition class. Every output unit $j$ performs the task of predicting the probability that the discriminator’s input is real within the domain $j$. Consequently, in our model, a generated image can be equally realistic for multiple domains which are not mutually exclusive. Based on the generator-discriminator interplay, we define the adversarial loss as:

3 $${L}_{{adv}}={{\mathbb{E}}}_{x,d}[\log {Di}{s}_{d}(x)]+{{\mathbb{E}}}_{x,{d}^{{\prime} },z}\left[1-\log {Di}{s}_{{d}^{{\prime} }}\left(G\left(x,{\hat{s}}^{{\prime} }\right)\right)\right]$$

where ${Di}{s}_{d}(x)$ stands for the ${d}^{{th}}$ output of the multi-task discriminator. Equation 3 essentially describes how the generator and the discriminator are jointly trained. The discriminator is tweaked to maximize the function ${L}_{{adv}}$ by learning to predict an observation ${x}_{i}$ as real in its domain ${d}_{i}$. Conversely, the generator strives to deceive its adversary by maximizing the probability that its generated output conditioned on ${\hat{s}}_{i}^{{\prime} }$ is labeled as a real input from the domain ${d}_{i}^{{\prime} }$.

The problem we are solving is a typical instance of distribution alignment, where the conditional distribution learned by the generator is matched to the real data distribution through a minimax optimization problem. To further push the generator network to associate an image to its domain-specific style, we train a style encoder ${E}_{{sty}}:{{\mathbb{R}}}^{{C}\times {H}\times {W}}\mapsto {{\mathbb{R}}}^{{S}}$ to approximate the style vector ${\hat{s}}_{i}^{{\prime} }$ starting from a transformed image ${\hat{x}}_{i}^{{\prime} }$. This is accomplished through the following loss function:

4 $${L}_{{sty}}={{\mathbb{E}}}_{x,d^{\prime} \!,z}[{||}\hat{s}^{\prime} -{E}_{{sty}}(G(x,\hat{s}^{\prime} ))|{|}_{1}]$$

Furthermore, to make sure the adversarial process preserves the domain-invariant characteristics of the input image, we push the model to reconstruct the original cell image from its transformed counterpart. To this end, we condition the decoding of ${\hat{x}}_{i}^{{\prime} }$ on the style vector ${E}_{{sty}}({x}_{i})$ to retrieve an approximation ${\hat{x}}_{i}$ of the original image ${x}_{i}$. Such a process further ensures that the style space learned by $f$ is in line with that implemented by ${E}_{{sty}}$ and it causes a compatible shift in the generated distribution. Let ${\hat{x}}^{{\prime} }=G(x,\hat{s}^{\prime} )$, we define the cycle consistency loss as follows:

5 $${L}_{{cyc}}={{\mathbb{E}}}_{x,d^{\prime},z}[{||x}-G\left({\hat{x}}^{{\prime} },{E}_{{sty}}\left(x\right)\right)|{|}_{1}]$$

Here, *x* is the source image before the transformation.

Finally, following prior work, we enforce diversity in the generated output by a style diversification loss. Given two independent random noise vectors *z*^(1)^ and *z*^(2)^ from the same standard normal distribution and the respective styles ${\hat{s}}^{{\prime} \left(1\right)}=f\left({e}_{d^{\prime} },{z}^{\left(1\right)}\right)$ and ${\hat{s}}^{{\prime} \left(2\right)}=f\left({e}_{{d}^{{\prime} }},{z}^{\left(2\right)}\right)$ for the same condition *d*′, we maximize the heterogeneity between their generated outputs through the following style diversification loss:

6 $${L}_{{ds}}=-{{\mathbb{E}}}_{x,{{d}^{\prime}},{{z}^{\left(1\right)}},{{z}^{\left(2\right)}}}[{||G}\left(x,{{\hat{s}}^{{\prime}{\left(1\right)}}}\right)-G\left(x,{\hat{s}^{{\prime} \left(2\right)}}\right)|{|}_{1}]$$

Equation 6 ensures that the random vector is not ignored, and a single condition can produce a distribution of diverse counterfactual responses.

Collecting all the terms together, the generator is trained to minimize the final loss:

7 $${L}_{{tot}}={\lambda }_{{adv}}{L}_{{adv}}+{\lambda }_{{sty}}{L}_{{sty}}+{\lambda }_{{cyc}}{L}_{{cyc}}+{\lambda }_{{ds}}{L}_{{ds}}$$

where the *λ* coefficients delineate scale parameters to define the relative importance of each component. All scaling terms are kept constant during training except for *λ*_*ds*_ which is decayed linearly across the budget of iterations until it reaches a value of 0.

Having depicted the optimization objective of our model, each training iteration proceeds through the following steps:

- An input $({x}_{i},{d}_{i})$ is sampled (always a control cell in the case of the perturbation prediction setting).
- A condition ${d}_{i}^{{\prime} }$ different from ${d}_{i}$ and two random vectors ${z}_{i}^{(1)}$ and ${z}_{i}^{(2)}$ are sampled from the respective distributions.
- The styles ${\hat{s}}_{i}^{{\prime} (1)}=f({e}_{{d}_{i}^{{\prime} }},{z}_{i}^{(1)})$ and ${\hat{s}}_{i}^{{\prime} (2)}=f({e}_{{d}_{i}^{{\prime} }},{z}_{i}^{(2)})$ are inferred and used to generate transformed inputs to approximate Eq. 6.
- The generated output ${\hat{x}}_{i}^{{\prime} }=G({x}_{i},{\hat{s}}_{i}^{{\prime} (1)})$ is used to approximate Eq. 3 together with the real batch.
- ${E}_{{sty}}$ computes style vectors from ${x}_{i}$ and ${\hat{x}}_{i}^{{\prime} }=G({x}_{i},{\hat{s}}_{i}^{{\prime} (1)})$ to approximate Eqs. 4 and 5.
- ${L}_{{tot}}$ is calculated as described in Eq. 7 and minimized via gradient descent.

#### Testing

Given an input observation $({x}_{i},{d}_{i})$ and a condition ${d}_{i}^{{\prime} }$ for which we would like to generate a prediction, IMPA addresses what morphological changes would be induced in the control image ${x}_{i}$ had it been drawn from ${d}_{i}^{{\prime} }$ instead of ${d}_{i}$. Provided the components described in the previous section, testing is carried out through the following steps:

- Sample ${z}_{i}\sim N(0,I)$ and collect ${e}_{{d}_{i}^{\prime} }$.
- Compute the style ${\hat{s}}_{i}^{{\prime} }=f({e}_{{d}_{i}^{\prime} },{z}_{i})$.
- Infer the content ${\hat{c}}_{i}=E({x}_{i})$.
- Perform decoding on ${\hat{{c}}}_{i}$ conditioned on the style vector ${\hat{s}}_{i}^{{\prime} }$ to obtain the result of the prediction ${\hat{x}}_{i}^{{\prime} }=D({\hat{c}}_{i},{\hat{s}}_{i}^{{\prime} })$.

In perturbation prediction, we choose as ${d}_{i}^{{\prime} }$ a treatment whose effect we wish to predict. When performing batch correction, we use IMPA to transport all images to a custom reference batch ${d}_{i}^{{\prime} }$ to remove technical variations between them.

### Architecture

#### Normalization method and style conditioning

##### Instance Normalization

Instance normalization^59^ (IN) differs from batch normalization^60^ (BN) in that it produces observation-specific scales and shifts instead of learning them for the whole batch. Given an observation ${x}_{{tchw}}$, where $t$ indexes the batch dimension, $c$ the channel, $h$ the height and $w$ the width, IN standardizes an observation as follows:

8 $${y}_{{tchw}}	=\frac{{x}_{{tchw}}-{\mu }_{{tc}}}{\sqrt{{{\sigma }_{{tc}}}^{2}+\epsilon }},\\ {\mu }_{{tc}}	=\frac{1}{{HW}}{\sum }_{l=1}^{H}{\sum }_{m=1}^{W}{x}_{{tclm}},\\ {\sigma }_{{tc}}	=\frac{1}{{HW}}{\sum }_{l=1}^{H}{\sum }_{m=1}^{W}{({x}_{{tclm}}-{\mu }_{{tc}})}^{2},$$

where ${\mu }_{{tc}}$ and ${\sigma }_{{tc}}$ are the pixel mean and variance of a single instance computed across spatial dimensions. $H$ and $W$ indicate the height and the width of the images, whereas ${y}_{{tchw}}$ is the outcome of the normalization. Since IN is implemented as a differentiable neural network layer, additional scaling and shifting parameters $\gamma$ and $\beta$ are learned during training, giving rise to the following expression:

9 $${IN}({x}_{{tchw}})={y}_{{tchw}}\cdot \gamma+\beta$$

Notably, Eq. 9 implements an affine transformation of the input.

##### Adaptive Instance Normalization (AdaIN)

IN has been demonstrated to normalize the input to a specific style controlled by learned affine transformation parameters. Intuitively, if we can learn to shift and scale the convolutional feature space of an observation based on a class-specific style, then we can implement a generator performing style transfer to a chosen target class. Thus, we employ ADAptive INstance Normalization (AdaIN)^34^ to perform domain translation in the decoder of our model.

Given an input $x$ and a style $y$, AdaIN computes the following transformation:

10 $${AdaIN}(x,y)=\sigma (y){IN}(x)+\mu (y)$$

where $\sigma (y)$ and $\mu (y)$ are learned style-dependent affine parameters that normalize the image $x$ to style $y$. By operating in the convolutional feature space, AdaIN facilitates domain transfer by enhancing the feature channels responsible for conveying a determined visual style response.

##### Residual block

All the image-processing components in our model are implemented as a residual block^61^. Any input $x$ is simultaneously fed to a convolutional stack called residual mapping and a shallow skip connection. Subsequently, the results from said branches are summed to produce the residual block output. An overview of the network layers in the residual block can be observed in Table 1. Encoder and decoder residual blocks differ in the way they modify the dimensionality in the spatial dimension. More precisely, the encoder network sequentially reduces the image height and width by a factor of two through average pooling, whereas the decoder upsamples feature maps spatially via nearest-neighbor interpolation. Since the residual mapping produces changes in dimensionality, the skip connection is equipped with average pooling or upsampling layers and a 1$\times$1 convolution to match the spatial and depth dimensions of the residual branch.

##### Generator

The architecture of the generator is illustrated in Table 2. It consists of an encoder and a decoder that implement the image translation task. The encoder computes the latent content starting from an image. Such a representation is high dimensional ($512\times 12\times 12$) to allow for the preservation of spatial features. Notably, the decoder network mirrors the encoder as shown in Table 2. While the encoder does not receive any information on the condition style, the decoder is conditioned on it via AdaIN. More formally, the convolutional features of the decoder residual block are scaled based on the condition style vector. To achieve condition-specific scaling of the residual blocks using AdaIN, the style vector $\hat{s}$ is passed through a tunable linear layer that approximates the functions $\sigma (\hat{s})$ and $\mu (\hat{s})$ and compute the affine transformation reported in Eq. (10).

##### Discriminator and style encoder

The architectures of the discriminator and style encoder are illustrated in Table 3. The discriminator is a convolutional neural network consisting of downsampling residual blocks and a final 2D convolutional layer with $3\times 3$ kernel. Let $p$ be the number of available conditions and $x$ an input with batch size $T$, number of channels $C$, width $W$ and height $H$. The discriminator acts on the input reducing it to an output array shaped $T\times p\times 1\times 1$. Successively, the spatial dimensions are trimmed and a sigmoid activation is applied to each node. The resulting $T\times p$ tensor represents the class-specific predictions for the input. The inferred style encoder has the same organization as the discriminator network. However, it projects an image onto a $T\times s$ tensor, where $s$ is the dimensionality of the style space. No activation function is applied to the output of the module.

##### Condition encoder

The condition encoder carries out a simple linear projection from the condition embedding to the approximated style space. Therefore, it is implemented as a simple fully-connected layer with no activation. When combining multiple types of treatment conditions, each perturbation type is represented by an embedding of different dimensionality. Therefore, instead of a single condition encoder, we derive multiple versions thereof, one per modality. All such condition encoders map into the same perturbation style space, which is used to induce a morphological change in control cells.

##### Gradient penalty

We control the magnitude of the discriminator’s updates by adding a gradient penalty term ${L}_{{reg}}$ with a coefficient ${\lambda }_{{reg}}$ to the loss. ${L}_{{reg}}$ computes the sum of squared gradients of the discriminator’s output with respect to all the pixels of the real input on which it is trained. The sum of squared gradients is then averaged across the batch dimension as follows:

11 $${L}_{{reg}}=\frac{1}{m}{\sum }_{i=1}^{m}{{||}{\nabla }_{{x}_{i}}{Dis}({x}_{i}){||}}_{2}^{2}$$

where ${x}_{i}$ is the *i*^*th*^ image of a real batch with *m* samples and ${\nabla }_{{x}_{i}}$ the gradient of the discriminator’s output with respect to the pixels of the input image *x*_*i*_.

##### Data augmentation

Each training image undergoes random vertical and horizontal flips with a probability of 0.3 before training as a form of data augmentation. Moreover, we add random noise elementwise to all images before feeding them to the encoder.

##### Weight initialization

To prevent the arising of vanishing and exploding gradients in the context of non-linear activation functions, we adopt the popular *He weight initialization*^62^. This method is specifically designed to stabilize training with the ReLU activation function and its variants by controlling the weight variance upon initialization. In practical terms, the He approach draws the initial weights w_*l*_ for layer *l* from the following distribution:

12 $${w}_{l}\sim N\left(0,\frac{2}{{n}_{l}}\right)$$

where ${n}_{l}$ is the number of input neurons of *l*. To fulfil the requirement of having zero-centred weights and outputs, the bias is set to 0 at the beginning.

### Baselines

#### StarGANv1 for perturbation prediction and batch equalization (Qian et al.^24^)

StarGANv1^41^ performs conditional image-to-image translation across multiple domains using a single adversarial network. Similar to IMPA, the architecture is based on an autoencoder model that maps input data to a latent space shared among domains. During training on a data point, a different condition is sampled as a one-hot encoded vector. Said vector is used to couple decoding by broadcasting and appending it to the input image as additional feature maps. Subsequently, the decoded output is passed to a multi-class discriminator that tries to simultaneously predict whether the result is real or generated and to which class it belongs. By learning to mislead the discriminator, the autoencoder acts as a generator of transformed images. As the generation is fully conditioned on a lookup table for the domain labels, the model is deterministic. The model presented by Qian et al.^24^ for batch equalization is also based on StarGANv1. The authors add additional losses to disentangle biological from technical features.

#### StarGANv2

StarGANv2^30^ upgrades the first version of the model from multiple perspectives. First, it involves a multi-task discriminator, similar to IMPA, rather than trying to discriminate images into different perturbation classes competitively. Moreover, the style space construction is different. To obtain the style used to apply a transformation, Gaussian noise is first sampled and passed through a condition-specific deep neural network, whose output is used to guide the decoding process by the generator. Using a neural network per condition makes the model unsuitable for translation tasks with a large set of target domains, such as high throughput screenings. StarGANv2 also differs from IMPA in that it has one style encoder’s head per condition rather than a single-headed neural network.

#### DMIT

Disentanglement for Multi-mapping Image-to-Image Translation (DMIT)^40^ performs multimodal and multi-domain image transformation via content and style disentanglement. It assumes that each image in the dataset is disentangled across three latent representations referred to as content, style and label spaces. Specifically, the image content and style are inferred through encoder networks, whereas the label is one-hot encoded and used for conditioning a residual decoder. The training is divided into a disentanglement and a translation path. The former pushes the model to reconstruct the original input from disentangled spaces and regularizes the style encoding to a multivariate normal distribution. Conversely, the translation path implements the adversarial mechanism where styles and domains are swapped, and a domain-conditioned discriminator is deceived into mislabeling generated images as real. What distinguishes DMIT from the other models is the presence of two latent regression terms that try to predict the inferred content and style vectors from a generated image.

#### DRIT + +

DRIT + + ^39^ encodes an image into two disentangled spaces: a domain-invariant content encoding and a condition-specific attribute vector. During training, random pairs of images from different domains are sampled and their attributes are swapped in the decoding process. From the decoded outputs, a multi-task generator tries to predict if the synthesized images are real and to what domain they belong. To force the content space to be completely class-agnostic, an additional content discriminator is deceived into failing to predict the domain of origin from the content vector. Moreover, a cross-cycle consistency loss is computed by training the model to invert the style exchange process. All steps are conditioned on one-hot domain encodings, which allow guiding the model to perform translation to a given class during inference.

#### Mol2Image

Mol2Image^21^ is a conditional generative model originally applied to the task of cell image generation. The authors implemented a multi-scale version of Glow, a generative framework based on normalizing flows. Cell images are encoded to multiple levels of latent representations at different scales obtained by decomposing the input images into coarse and fine-grained downsampled representations via a Haar wavelet image pyramid. The latent codes are regularized to a Gaussian distribution whose parameters are conditioned on the next level of the pyramid and a dense drug representation is obtained via Graph Neural Networks (GNNs). To further enhance drug conditioning, the authors train the model with a contrastive loss, which pulls the latent representations of images closer to the embedding of the drug used to perturb them. In the generation phase, training and unseen drugs are used to condition the sampling of latent codes for image synthesis, therefore providing a tool to infer the morphological effects of drug perturbations on cell data.

#### PhenDiff

PhenDiff is a diffusion-based^63^ model converting control cells into their perturbed version. It was tested originally on the BBBC021 dataset. The model is trained to generate cells from noise conditioned on perturbation attributes via a diffusion model trained to learn the inversion of a noising process mapping data to a standard Gaussian distribution. During inference, control images are fed to the forward noising process, converted into images from the prior and then decoded using the condition attribute of the target perturbation. Generation requires simulating a Stochastic Differential Equation (SDE) discretized across multiple time steps.

#### StyTR2

The StyTR2^42^ model utilizes a transformer-based architecture for image style transfer with a structured pipeline. The process begins by splitting the content and style images into patches and applying a linear projection to obtain patch sequences. The content sequences are fed into a content transformer encoder, while the style sequences are processed by a style transformer encoder. Following the encoders, a multi-layer transformer decoder is employed to stylize the content sequences based on the style sequences. The final output is produced using a progressive upsampling decoder, which reconstructs the stylized image from the transformed sequences.

#### Untransformed images

Alongside neural network baselines, we report evaluation scores computed on untransformed control images. In other words, we compare the source images before translation with the true perturbed examples from the dataset. This provides a lower bound on the model performance as it represents the evaluation score, we would expect if the network could not translate the inputs at all.

### Evaluation and metrics

#### Fréchet Inception Distance (FID)

The Fréchet Inception Distance (FID)^43^ measures the difference between the real and generated image distributions in the feature space learned by an Inception V3 model^64^ pre-trained on the ImageNet dataset^65^. Batches of true and fake samples are passed through the model and 2048-dimensional encodings are extracted from the last pooling layer. This produces visually relevant feature vectors that are used as a proxy to define the perceptual distance between the compared distributions. Specifically, for both real and generated batches, the mean and the covariance of the derived encodings are calculated. Let *m*_*r*_ and *m*_*g*_ be the mean Inception V3 embedding vectors for real and generated images and *C*_*r*_ and *C*_*g*_ be the relative covariance matrices. We want to compare the feature distribution heterogeneity between true and fake images through the Frechét distance metric. To this end, we assume that both real and generated inception features are distributed as multivariate normal distributions and that their means and the variances can be used to compute the Frechét distance between them. As a result, the FID is expressed as:

13 $${d}^{2}(({m}_{g},{C}_{g}),({m}_{r},{C}_{r}))={\left|\left|{m}_{g}-{m}_{r}\right|\right|}_{2}^{2}+{Tr}({C}_{g}+{C}_{r}-2{({C}_{g}{C}_{r})}^{1/2})$$

where Tr refers to the trace of a matrix and ${{||}{\cdot }{||}}_{2}^{2}$ is the squared *L*^*2*^ -norm. To make it comparable with the other scores, we report the inverse of the FID score.

#### Coverage

FID produces a summary of the distance between two distributions and does not separately evaluate the fidelity and diversity of the generated sample to the real data^44^. To address this, we introduce the Coverage metric to evaluate our generative model.

Let *X* and *Y* be respectively samples of real and generated images. We approximate an image manifold as:

14 $${manifold}({x}_{1},\ldots,{x}_{N})={\cup }_{i=1}^{N}B\left({x}_{i},{NN}{D}_{k}\left({x}_{i}\right)\right)$$

where $B(x,d)$ is the sphere defined around the point *x* with radius *d* and ${NN}{D}_{k}(x)$ is the distance of the furthest nearest neighbor from *x* in a neighborhood of size *k*. Given a manifold around the true data *X*, we measure coverage:

15 $${Coverage}=\frac{1}{N}{\sum }_{i=1}^{N}{1}_{\exists \, {j \, \, s}.t \, {y}_{j}\in B\left({x}_{i},{NN}{D}_{k}\left({x}_{i}\right)\right)}$$

Coverage quantifies the extent to which generated samples cover the entire real sample space, with higher values indicating that more real observations have corresponding generated examples in their neighborhood.

#### Accuracy of MoA classification

We additionally evaluate model predictions for drug perturbations based on the frequency with which they are correctly labeled with their actual MoA by a pre-trained classifier. More specifically, we train a classifier with the same architecture as the model’s discriminator (see the Architecture section) to predict MoA labels on the true images. Subsequently, we transform control cells into their drug-perturbed counterparts and infer the MoA on the generated images using our classifier. The higher the accuracy with which a model’s prediction is labeled with its true MoA, the better the generation output.

#### Tanimoto similarity

Tanimoto similarity is a metric commonly used in cheminformatics to compare the similarity between two molecular fingerprints. Molecular fingerprints are a way to represent the structure of molecules in a binary format, where each bit represents the presence or absence of a particular structural feature or substructure within the molecule. The Tanimoto similarity coefficient is calculated as the ratio of the intersection of the fingerprints to the union of the fingerprints:

15 $$T=\frac{|A\cap B|}{|A\cup B|}$$

Where:

- ${|A}\cap {B|}$ is the number of features (bits) present in both fingerprints A and B.
- ${|A}\cup {B|}$ is the total number of features (bits) present in either fingerprint A or B.

This coefficient ranges from 0 to 1, where 0 indicates no similarity and 1 indicates identical fingerprints.

#### Evaluation of morphological features

We used the CellProfiler 4.2.1^38^ software to calculate reliable morphological features of both generated and real images from the test set for comparison. Through this, we aim to evaluate if the transformations produced by our model on control images reproduce the phenotypic shift caused by a perturbation. A custom CellProfiler script is executed on the dedicated graphical user interface to perform segmentation and extract phenotypic profiles for cells. Briefly stated, the software identifies cell nuclei via Otsu thresholding^66^ and uses them as seeds to outline the corresponding objects in the cytoplasm channels via Watershed segmentation^67^. The outcome of this step is used to calculate an array of features quantifying cell shape, size, intensity distribution and texture across high-content channels. In total, we obtain 356 morphological features per image.

#### Feature importance calculation

To evaluate the quality of the transformations computed by IMPA from an image analysis perspective, we first define the most important features for discriminating between controls and single perturbation types on the real data. Concretely, importance scores are derived by running a Random Forest algorithm classifying between control and compound-stimulated cells for all the evaluated perturbations using the morphological features as input. The algorithm is executed 10 times, and feature importance is estimated as the out-of-bag score derived from the Random Forest algorithm across 500 trees. The most important features according to the analysis are assumed to be those that change significantly between controls and treated cells. If such features present a similar distribution in IMPA’s generations as in the real data, then our image translation framework successfully models the phenotypic shift from the controls.

#### Average Silhouette Width (Silhouette batch)

ASW relates distances within a cluster with those between clusters to evaluate separation between groups indicated by a label. The score is normalized to a scale of 0 to 1. Since the batch labels are used to define the clusters, the ASW score can be used to quantify batch mixing after batch correction comparing intra and inter-batch distances between equalized observations.

#### iLISI

The iLISI score, proposed to evaluate batch mixing, is computed from neighborhood lists per node in integrated kNN graphs of the data. It utilizes the inverse Simpson’s index to determine the number of cells drawn from a neighborhood list before encountering a duplicate batch. This score ranges from 1 to the total number of batches in the dataset, representing perfect separation and perfect mixing, respectively. In the scIB metrics package^57^, the score is normalized between 0 and 1.

#### Entropy and Gini index

Entropy and Gini index are used to evaluate the batch mixing within clusters obtained from image features. Clusters were computed using the Leiden algorithm^56^ implementation in the Scanpy^68^ package with default parameters. As measures of impurity, the higher the values of entropy and Gini index per cluster with respect to batch labels, the higher the mixing of batches in the chosen representation space. We perform clustering using image features extracted with a ViT. For each cluster, we compute the frequency of all *k* batches $\{{p}_{1},\cdots,{p}_{k}\}$. Then, the batch entropy for the cluster *i* is:

$${H}_{i}=-{\sum }_{j=1}^{k}{p}_{j}{lo}{g}_{2}({p}_{{ij}})$$

and the Gini index is:

$${G}_{i}=1-{\sum }_{j=1}^{k}{{p}_{{ij}}}^{2}$$

Here, the index *j* indicates the batch and *p*_*ij*_ is the frequency of batch *j* in cluster *i*. The reported scores are the average entropy and Gini index across clusters.

### Dataset description

#### BBBC021

We choose the BBBC021v1 dataset^36^ from the Broad Bioimage Benchmark Collection. It contains images from a high-content screening assay featuring p53 wild-type MCF-7 cell lines of breast cancer. In the experiment, each well is fluoresced across three channels: nucleus (stained with DAPI), *β*-tubulin (marked with an anti-*β*-tubulin antibody) and F-actin (stained with Phalloidin). Precisely, the assay includes a series of 55 96-well plates grouped into batches acquired over ten weeks. Each plate contains six negative controls treated with dimethyl sulfoxide (DMSO) only and six positive controls incubated with Taxol. The remaining wells are exposed to active compounds in triplicates and at 8 different concentrations for 24 hours. The original dataset reports the screening of 112 molecules. A popular subset of the dataset was annotated in Ljosa et al.^12^, where only 38 perturbations are reported based on their potential to induce a phenotype at the used concentrations. In general, 12 MoAs were annotated in the 38-drug subset. From this group, the authors claim that only 6 MoAs were visually distinguishable in the images, whereas the rest were mined from the literature. The dataset consists of 2,528 full-well images with a resolution of 1024×1280 pixels each.

#### RxRx1

The RxRx1^54^ dataset involves 16-bit fluorescence microscopy images of cell lines perturbed by 1,138 distinct small interfering RNAs (siRNA). Fluorescent images were obtained through the Cell Painting assay across six channels highlighting heterogeneous cellular compartments. Four different cell lines (HUVEC, RPE, HepG2, and U2OS) were analyzed in distinct numbers of batches grouping 384-well plates. In total, 51 experimental batches were produced, with HUVEC being the most frequent cell line. The recovered images were downsampled to a resolution of 512×512 pixels to support downstream applications.

#### cpg0000

The JUMP-cpg0000 dataset^58^ consists of three perturbation types: CRISPR, ORF overexpression, and compound treatments. For most of the 160 target genes designed, two sister compounds and CRISPR guides are included, while only one type of ORF per gene is present. The JUMP-cpg0000 dataset contains images and profiles of cells perturbed separately by chemical and genetic perturbations. Chemical perturbations involve small molecules (compounds) affecting cell function, while genetic perturbations include ORFs overexpressing genes and CRISPR guides mediating CRISPR-Cas9 knockout of gene function. Each gene is targeted by one ORF, two CRISPR guides (except for 15 genes), and one or two compounds thought to influence the function of the gene’s product. These compounds are selected from Broad’s Drug Repurposing Hub dataset, which comprises FDA-approved drugs, clinical trial drugs, and pre-clinical tool compounds. In total, the JUMP-cpg0000 dataset comprises 34,336 well images across 10 plates. Imaging was carried out by the Cell Painting assay^7^ across 5 channels. The negative controls included in the JUMP-cpg0000 dataset for each perturbation modality are the following: DMSO-treated controls for compounds, 15 ORFs with the weakest signature in previous image-based profiling experiments for ORFs, and 30 guides targeting an intergenic site or non-existing target sequences in human cells for CRISPR. Additionally, positive controls are included in the dataset.

### Dataset pre-processing

#### Illumination correction algorithm and normalization

To reduce illumination artefacts due to plate effects, we perform the illumination correction algorithm described in Singh et al.^69^ on full-resolution images. First, images from each plate are aggregated and used to compute a flatfield image by taking the pixelwise 10th percentile within each plate group. Successively, the result is smoothed out by applying a Gaussian filter with a standard deviation of 50. The pixels of each well are divided by the flatfield image of the respective plate to yield the background intensity distribution for adjustment. Furthermore, the dynamic range of each image is enhanced by clipping the pixel values at 1 and computing the natural logarithm of the intensities. Following the original implementation, we additionally clip the transformed pixel intensities at a maximum of 5 to avoid outliers. The output is linearly re-scaled between 0 and 255 and quantized to 8 bits.

#### BBBC021 single cells

The full-resolution images are corrected for illumination and cropped into patches centred around single nuclei in squares of 96×96 pixels. To this end, we use the coordinates of 454,793 cells pre-computed by Ljosa et al.^12^ and exclude objects at less than 48 pixels from the image borders. We ruled out drugs with undisclosed chemical structures. To remove corrupted images displaying empty wells or pixel noise, we empirically filtered out all the images with a pixel variance lower than 2000. Moreover, we reduced the number of highly blurred images in the dataset by computing a normalized perceptual blur metric^70^ over each crop, where 1 indicates maximal blur and 0 absence thereof. A threshold for acceptance of an image was set at 0.65 to avoid over-pruning while still filtering excessively low-quality wells. Alongside the complete data, we created a reduced version of the dataset for comparison with the baseline image-to-image translation architectures. Specifically, we selected Cytochalasin B, Taxol, Vincristine, AZ258 and AZ138 inducing actin disruption, tubulin destabilization, tubulin stabilization, Aurora kinase inhibition and Eg5 inhibition, respectively. The drug for each MoA was manually selected based on image quality and effect size. The subset version of the dataset contains 20,313 images.

#### BBBC021 large field of view

We additionally created a version of the datasets consisting of larger patches with size 256 × 256, resized to a spatial resolution of 128 pixels. We used the larger field of view for the experiments reported in Fig. 2. Here, we kept all the drugs with available structures, resulting in a dataset with 99 compounds and more than 118k images. Illumination correction was carried out as in the single-cell BBBC021 dataset.

#### RxRx1

The images of RxRx1^54^ were corrected for illumination effects within the plates and their nuclear channels were segmented via Otsu thresholding. We derived patches of dimension 96 × 96, including one or a few cells depending on the bulkiness of the well. For simplicity, we focused on the U2OS cell line of osteosarcoma as our model does not account for inter-cell line transformations. In total, we obtained more than 170k images for 1,139 conditions (1,108 treatments, 30 control treatments and 1 untreated example per plate). Finally, we partitioned the dataset into 80% training and 20% test sets.

#### JUMP-cpg0000

We first performed plate-based illumination correction on the images following the pipeline described in Singh et al.^69^ The images were quantized to 8 bits. Image patches used for training were derived using the cell centres published by the authors^58^ and subsampled to 700,000 images initially. Around each cell, patches of side length 256 were derived and resized to  96 × 96. Images centered at cells located on the border of the view were discarded. We further subset the conditions to the 1,070 perturbations whose target genes are included in the Gene2Vec model. The total number of images arising from this procedure is 435,160.

### Perturbation embedding

#### Drug compounds in BBBC021

As drug embeddings in BBBC021, we employ Morgan Fingerprints^31^, binary chemical features computed by the RDKit Python package. Every compound with a disclosed chemical structure can be associated with a 1024-dimensional descriptor vector computed directly from its SMILES representation. These fingerprints capture both local and global structural features, enabling efficient compound comparison and prediction tasks in cheminformatics and drug discovery. To preserve their physiochemical meaning, the drug embeddings are made non-trainable.

#### Drug, CRISPR and ORF embeddings in JUMP-cpg0000

In the JUMP-cpg0000 experiment drugs were embedded as the concatenation between their associated Morgan Fingerprints and a Gene2Vec^32^ embedding of their target genes. The latter encoding is devised to obtain a shared embedding portion across perturbation types. The CRISPR embeddings were, instead, derived as concatenations of the corresponding Gene2Vec embedding and a dense representation of the guide nucleotide sequence via HyenaDNA^33^. A similar logic was applied to ORF embeddings, but instead of a guide sequence, we featurized the 5’ sequence flanking the ORF. The final drug embeddings are of dimensionality 1224, the CRISPR embeddings 328 and the ORF embeddings 456. The size mismatch between ORF and CRISPR derives from the difference in sequence length between the CRISPR guide and the 5’ ORF flanking region. For the former, we use a HyenaDNA model trained on 1k sequence length. For the latter, we use the same model trained on a sequence length of a maximum of 32k bases.

#### Batch correction

When using IMPA for batch correction, each condition is represented by an embedding trained alongside the model.

#### Additional aspects

Chemical structures in the figures were drawn with the PubChem sketcher^71^ software.

### Reporting summary

Further information on research design is available in the Nature Portfolio Reporting Summary linked to this article.

## Supplementary information

Supplementary Information

Reporting Summary

Peer Review file

## Source data

Source data

## Data Availability

The data included in the present study is publicly available. Specifically, we utilized the BBBC021 [https://bbbc.broadinstitute.org/BBBC021], the RxRx1 [https://www.rxrx.ai/rxrx1] and the cpg0000 [https://cellpainting-gallery.s3.amazonaws.com/index.html] datasets. Pre-processed versions of the datasets and model checkpoints have been deposited on Zenodo under accession code 8307629. Source data are provided with this paper.

## Code availability

The code for our model and additional links to data and checkpoints are available at https://github.com/theislab/IMPA^72^.
