# Supplementary material for: Predicting cell morphological responses to perturbations using generative modeling
Source: Nat Commun. 2025 Jan 8;16:505. doi: 10.1038/s41467-024-55707-8 (PMC11711326; doi:10.1038/s41467-024-55707-8)
Supplement: Supplementary file 1 — Supplementary Information [file 41467_2024_55707_MOESM1_ESM.pdf]

Supplementary information

Figures

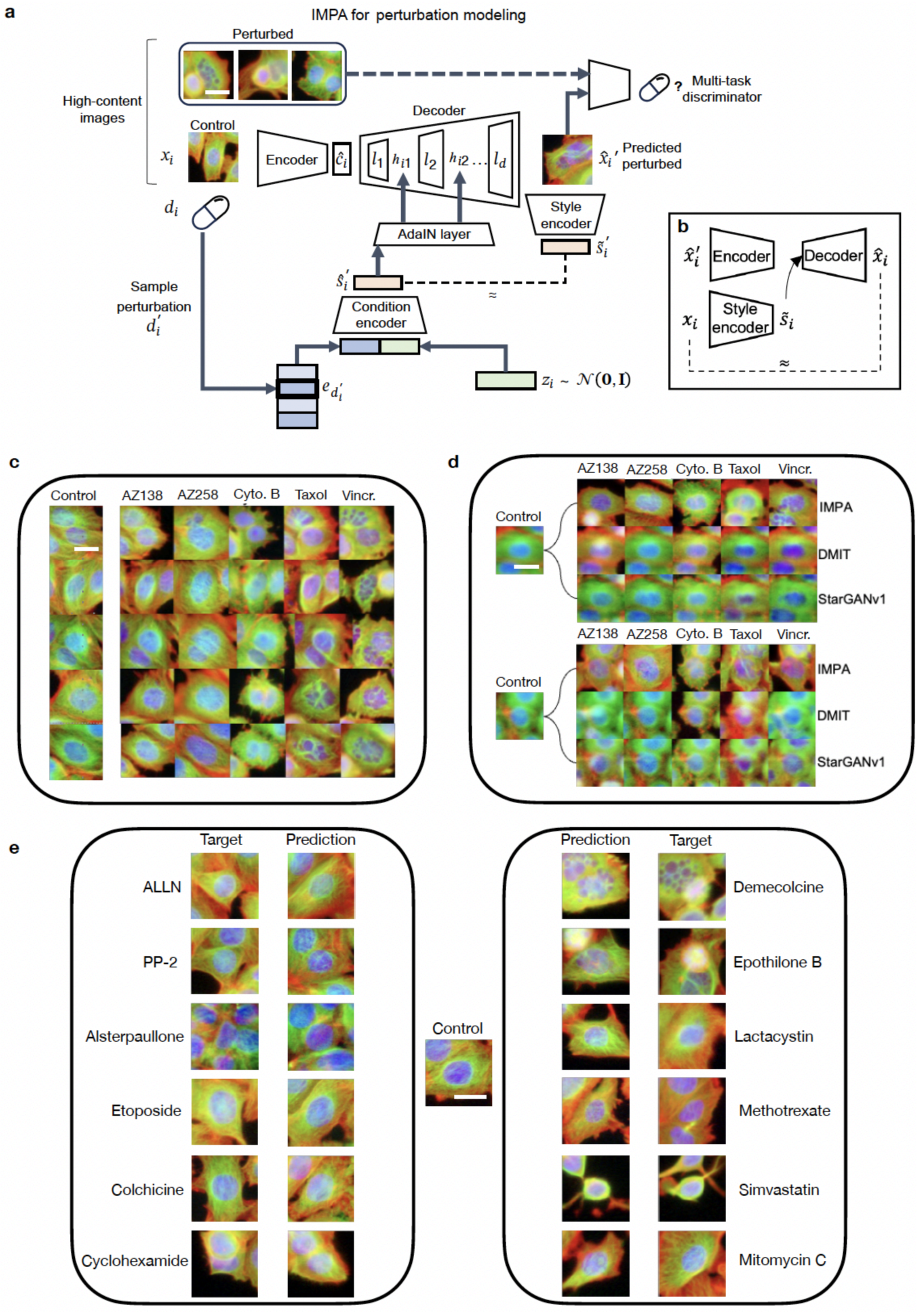

Supplementary Figure 1 | Model architecture and additional results for IMPA on BBBC021. (a-b) Depiction of the model's architecture similar to Fig. 1. The model is additionally equipped with a cycle consistency loss which reconstructs the input image conditioned on the style of the transformed one. The scale bar is 20 μm**. (c)** Additional predictions on the 5-drug version of the BBBC021 dataset. Vincr. and Cyto. B are abbreviations for Vincristine and Cytochalasin B. The scale bar is 20 μm**. (d)** IMPA as a denoising tool. The model converts corrupted control images into their denoised perturbed versions. Vincr. and Cyto. B are abbreviations for Vincristine and Cytochalasin B. The scale bar is 20 μm**. (e)** Additional examples of IMPA's prediction on the whole BBBC021 dataset next to real examples of perturbed cell images. The scale bar is 20 μm**.**

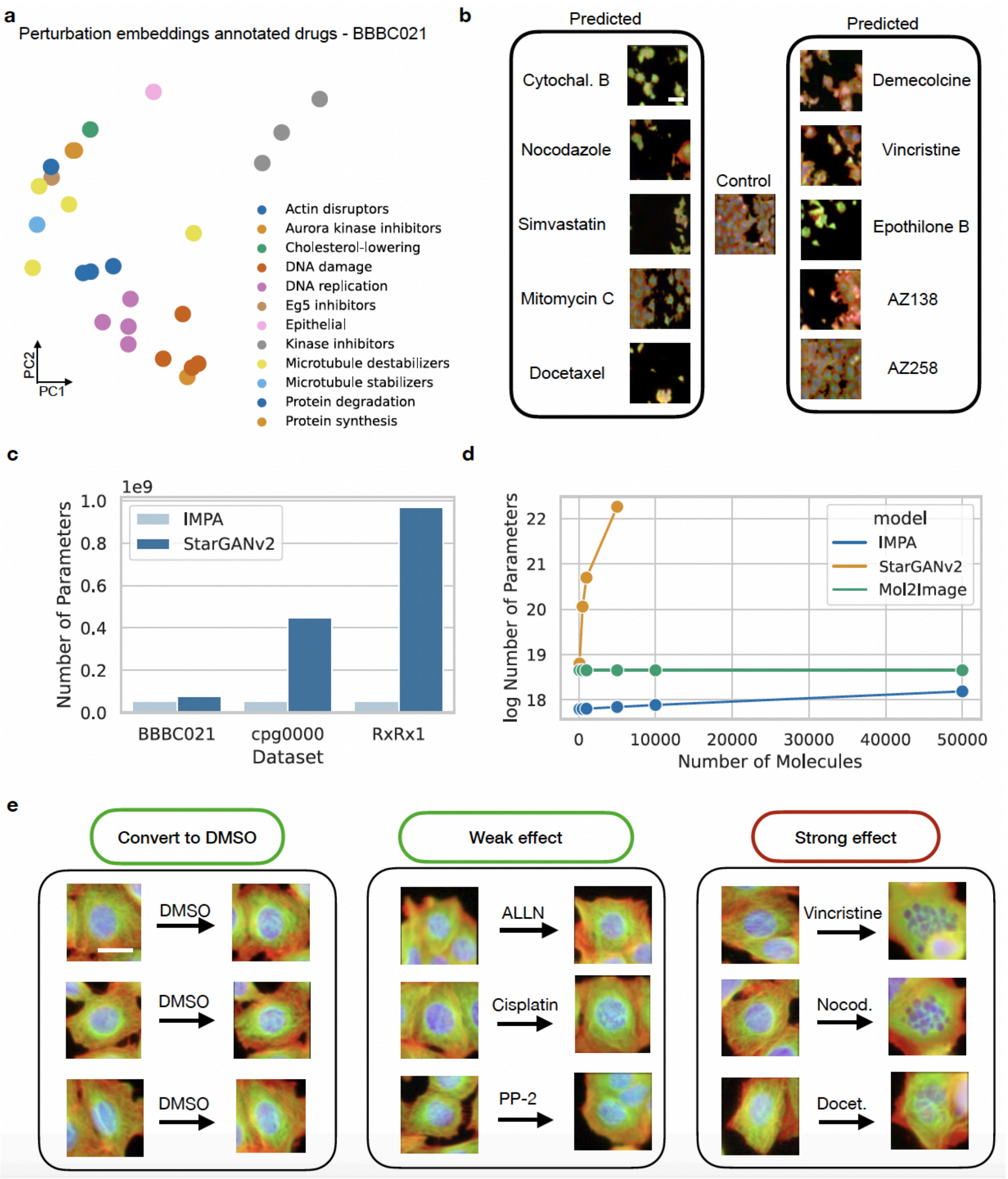

Supplementary Figure 2 | Additional results on whole slide predictions, scaling properties and variable effect size prediction using BBBC021. (a) PCA plot of the perturbation space learnt by IMPA on the annotated portion of the BBBC021 dataset. **(b)** Additional examples of predictions of drug effects on images larger fields of view. Cytochal. B is an abbreviation for Cytochalasin B. The scale bar is 30 μm. **(c)** Comparison between IMPA and StarGANv2 based on the number of parameters across different datasets. Source data are provided as Source data files. **(d)** Comparison between IMPA, Mol2Image and StarGANv2 in terms of numbers of parameters as a function of the number of perturbations in the dataset. Beyond 5000 perturbations StarGANv2 does not fit into memory, hence the interrupted plot. Source data are provided as Source data files. **(e)** Qualitative performance of IMPA when transforming controls into DMSOs, drugs with weak effects and drugs with strong effects. Docet. and Nocod. stand for Docetaxel and Nocodazole. The scale bar is 20 μm.

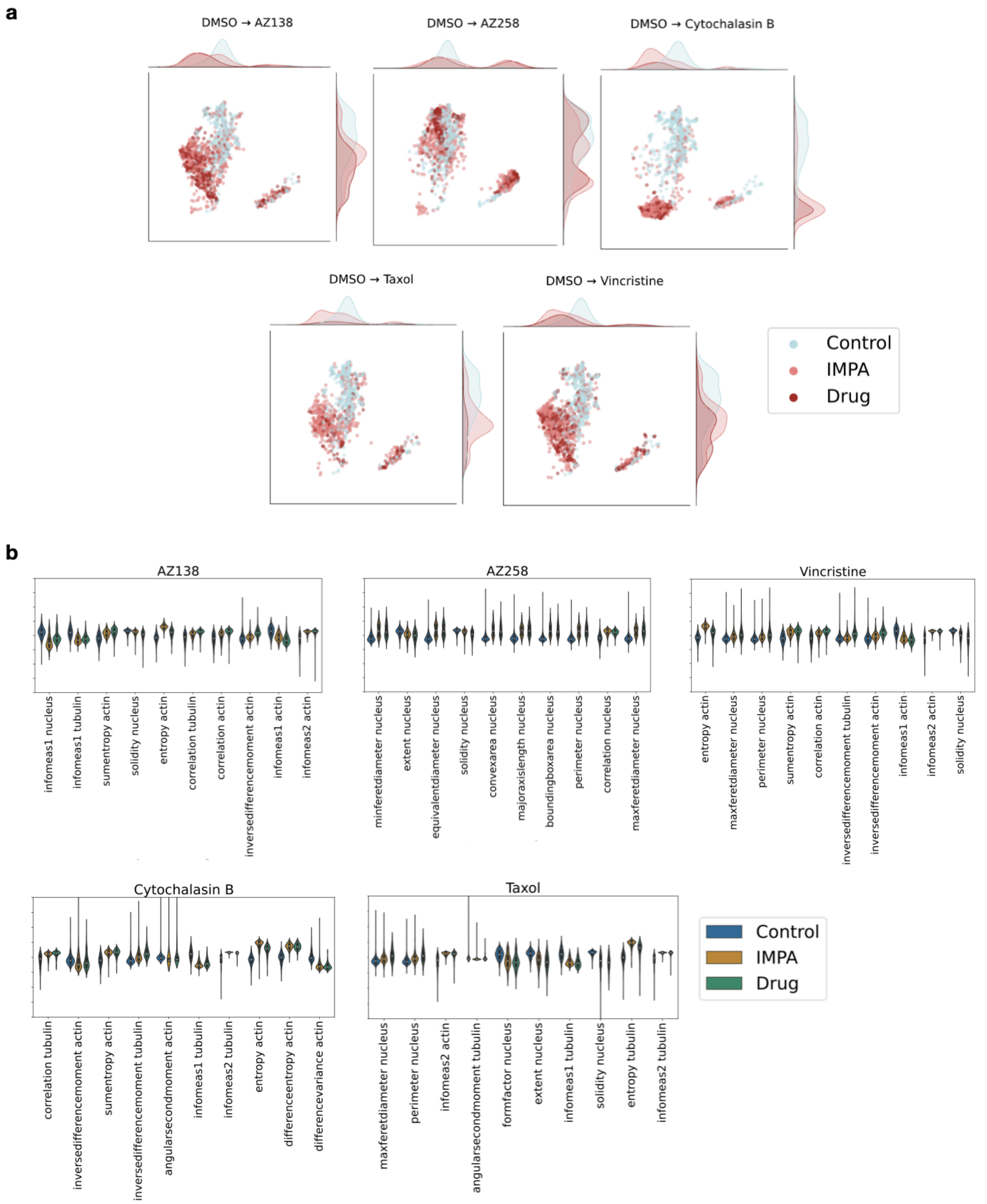

Supplementary Figure 3 | Morphological feature analysis with IMPA on BBBC021. (a) 2D UMAP plots of 356 CellProfiler features before and after transformation with IMPA for five drugs. Data points represent individual control, transformed control and real perturbation images in the test set of BBBC021. dataset. **(b)** Distribution of the ten most important discriminative features in controls, IMPA's predictions and true perturbation images for five drugs in BBBC021. The violin plots' boxes show the feature distribution quartiles, with whiskers marking the 95% quantiles. Source data are provided as Source Data files.

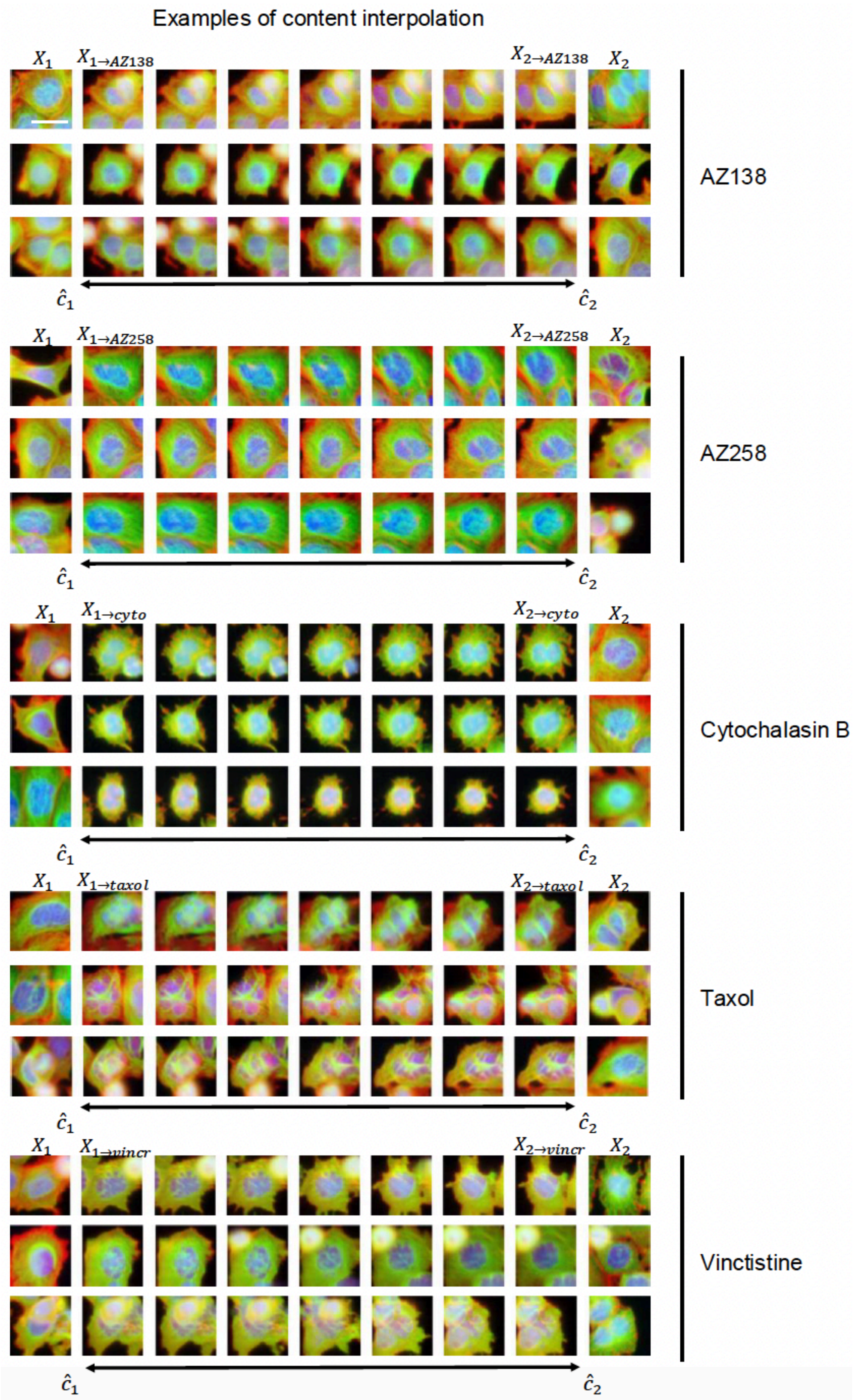

Supplementary Figure 4 | Content interpolation examples on BBBC021. Content interpolation of images of cells with constant styles. X_1 and X_2 are perturbed via IMPA by a chosen drug and their content encodings are interpolated to yield intermediate generated cells with fixed style. The scale bar is 20 µm.

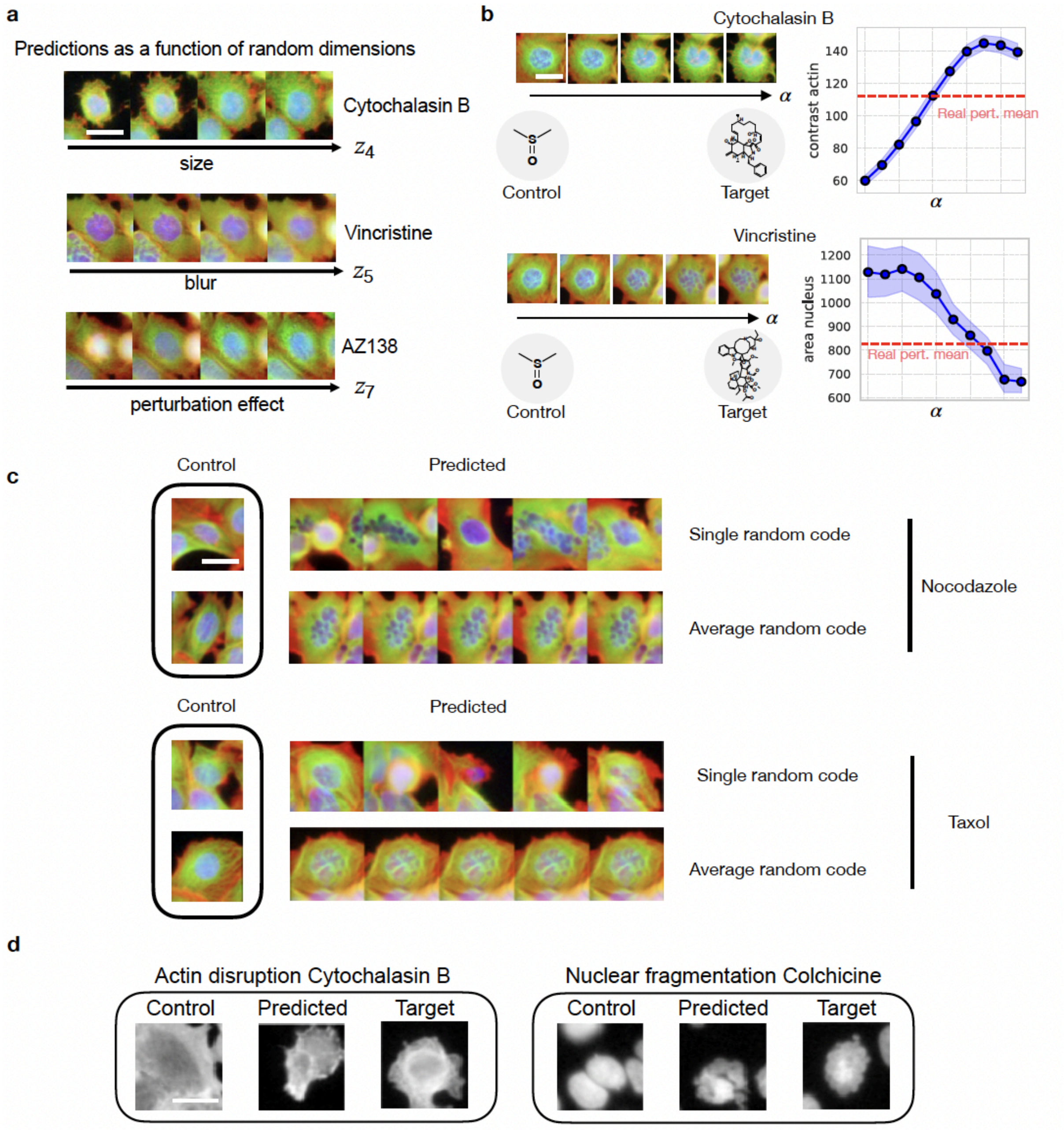

Supplementary Figure 5 | Interpolations and interpretability on BBBC021. (a) Relation between generated morphological responses to drugs and single dimensions of the random Gaussian conditioning vector. The scale bar is 20 μm. **(b)** Left - Style interpolations between DMSO (control) and the drugs Vincristine (tubulin destabilizer) and Cytochalasin B (actin disruptor). The parameter α represents the amount of perturbation added to the DMSO style under the formula $(1-\alpha)s_{\mathrm{DMSO}} + \alpha s_{\mathrm{drug}}$, where s stands for style. Right - The gradual change of the most drug-impacted morphological features along the interpolation from control to perturbation style for Vincristine and Cytochalasin B. Data are presented as mean ± 95% CI. Source data are provided as Source data files. The scale bar is 20 μm. **(c)** Predictions of drug responses with single random styles and random styles averaged over 100 independent draws for Nocodazole and Taxol. The scale bar is 20 μm. **(d)** Prediction of actin disruption and nuclear fragmentation due to apoptosis in Cytochalasin B and Colchicine, two drugs held out from training on BBBC021. The scale bar is 20 μm.

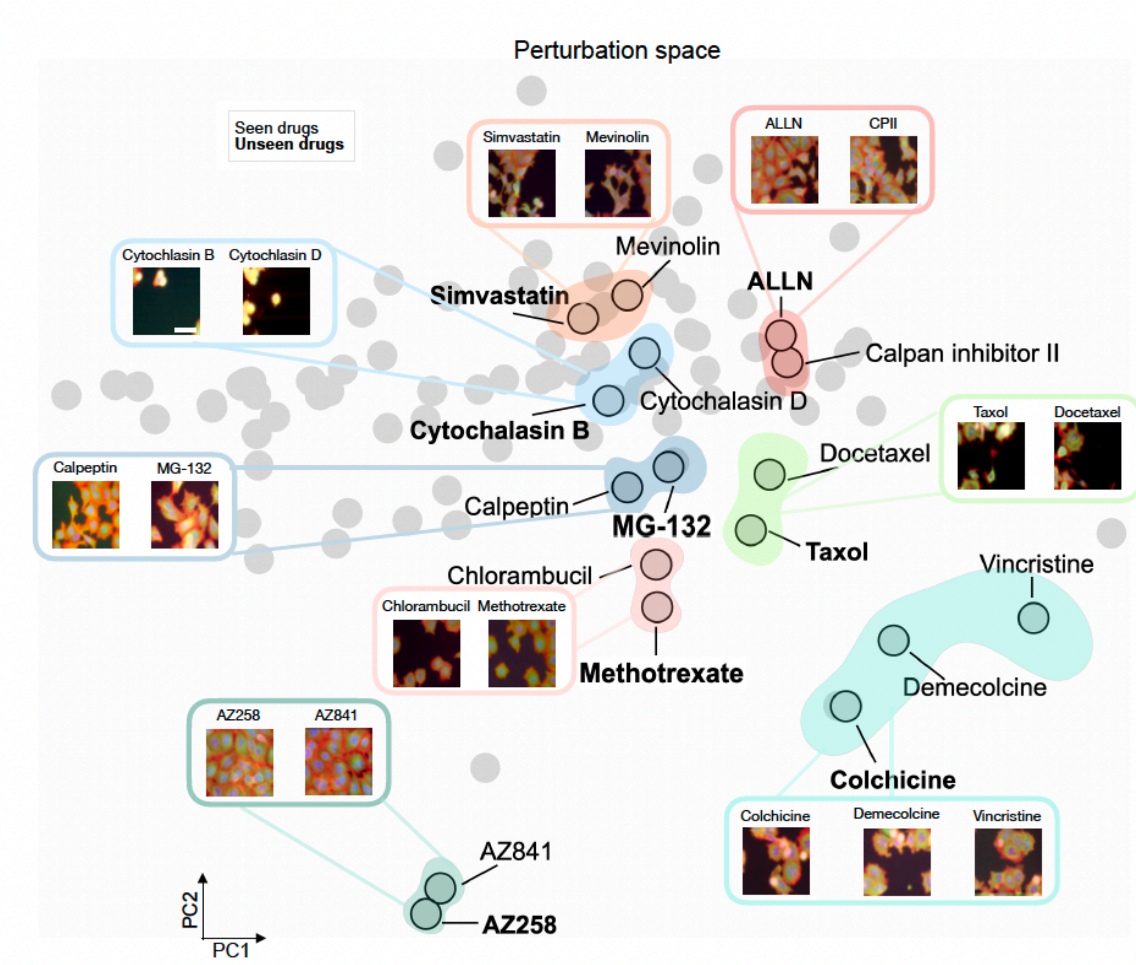

Supplementary Figure 6 | Visualization of real perturbation images in the drug space of BBBC021. 2D PCA plot of the perturbation space learned by the style encoder. Perturbations highlighted in bold are part of the set of held-out compounds. Groups are highlighted as drugs triggering a similar phenotypic effect in the original dataset. Examples of real perturbation images for different perturbations in each group are annotated on the plot. The scale bar is 20 µm.

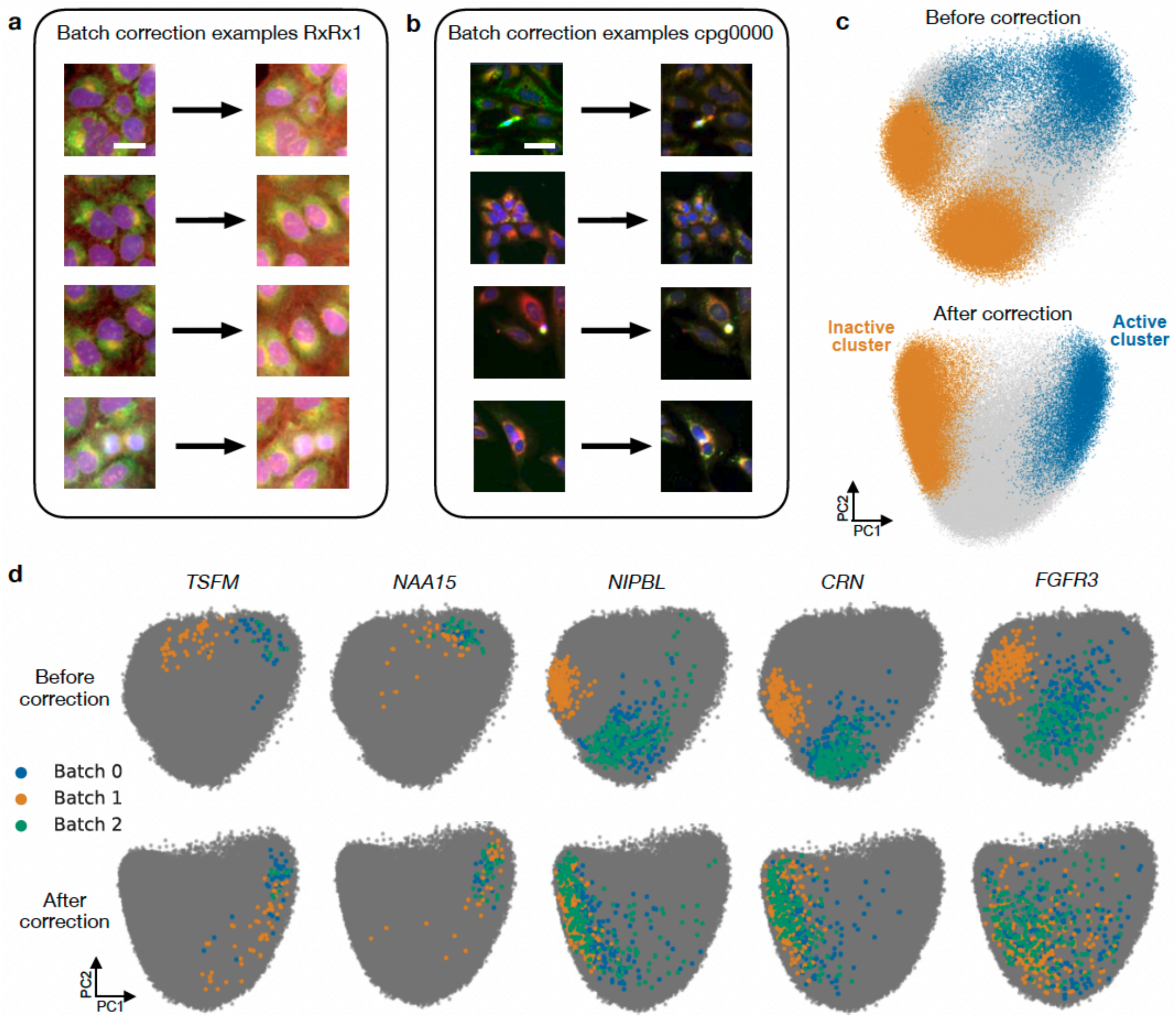

Supplementary Figure 7 | Additional results on the batch correction task. (a-b) Examples of batch correction acting on illumination differences carried out by IMPA on RxRx1 and cpg0000. The scale bars are 20 µm (a) and 30 µm (b)**. (c)** Active and inactive clusters of genetic perturbations visualized on the PCA plots of image features extracted before and after correction with IMPA from RxRx1. Features were computed by a pre-trained Cell Painting ViT. **(d)** Highlighted cell image features before and after correction by IMPA colored by batch for controls and treated with siRNAs targeting five genes.

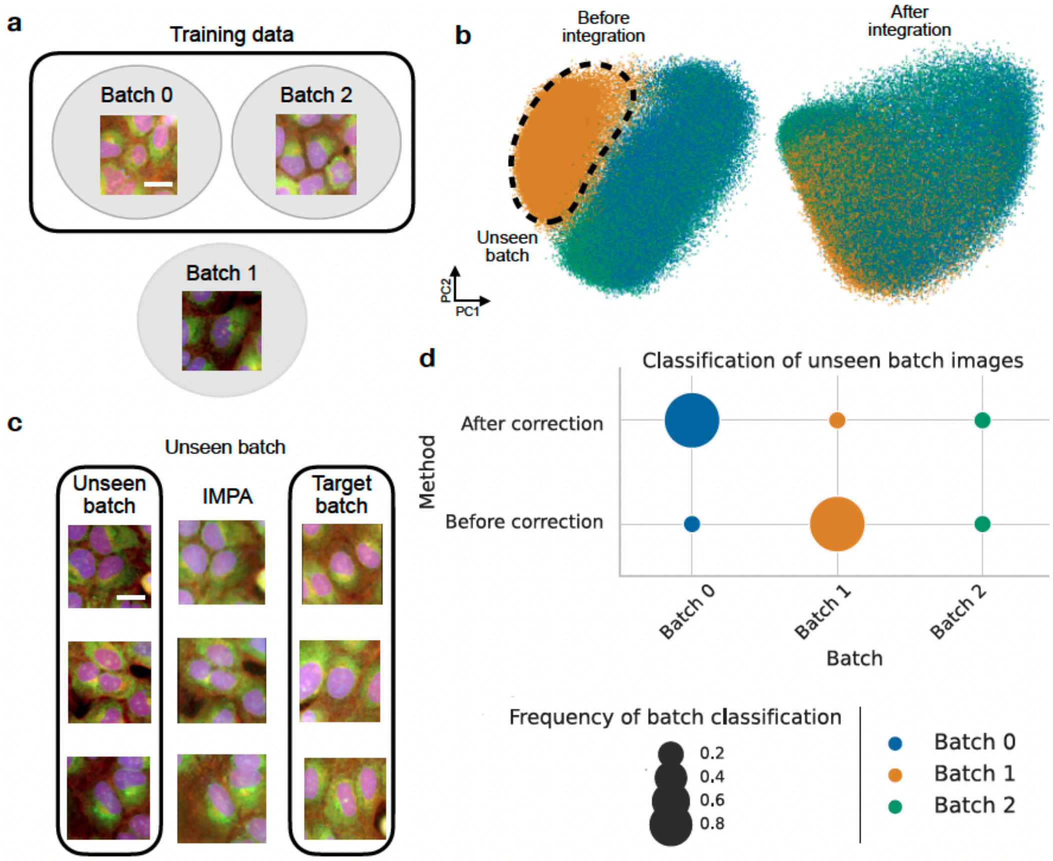

Supplementary Figure 8 | Correction performance on unseen batches on RxRx1. (a) Depiction of the held-out batch experiment. IMPA is trained on two batches (0 and 2) and used to predict a left-out batch (1). The scale bar is 20 µm. **(b)** ViT features colored by batch before and after correction. **(c)** Visual depiction of batch effect results obtained by IMPA on images from an unseen batch. Left - starting image from the source batch. Centre - Transformation by IMPA to reference batch. Right - Example of images from the target batch. The scale bar is 20 µm. **(d)** Classification of images from the unseen batch 1 before and after transformation to batch 0. The classification is carried out by a classifier trained to recognize batches on the real images. Source data are provided as Source data files.

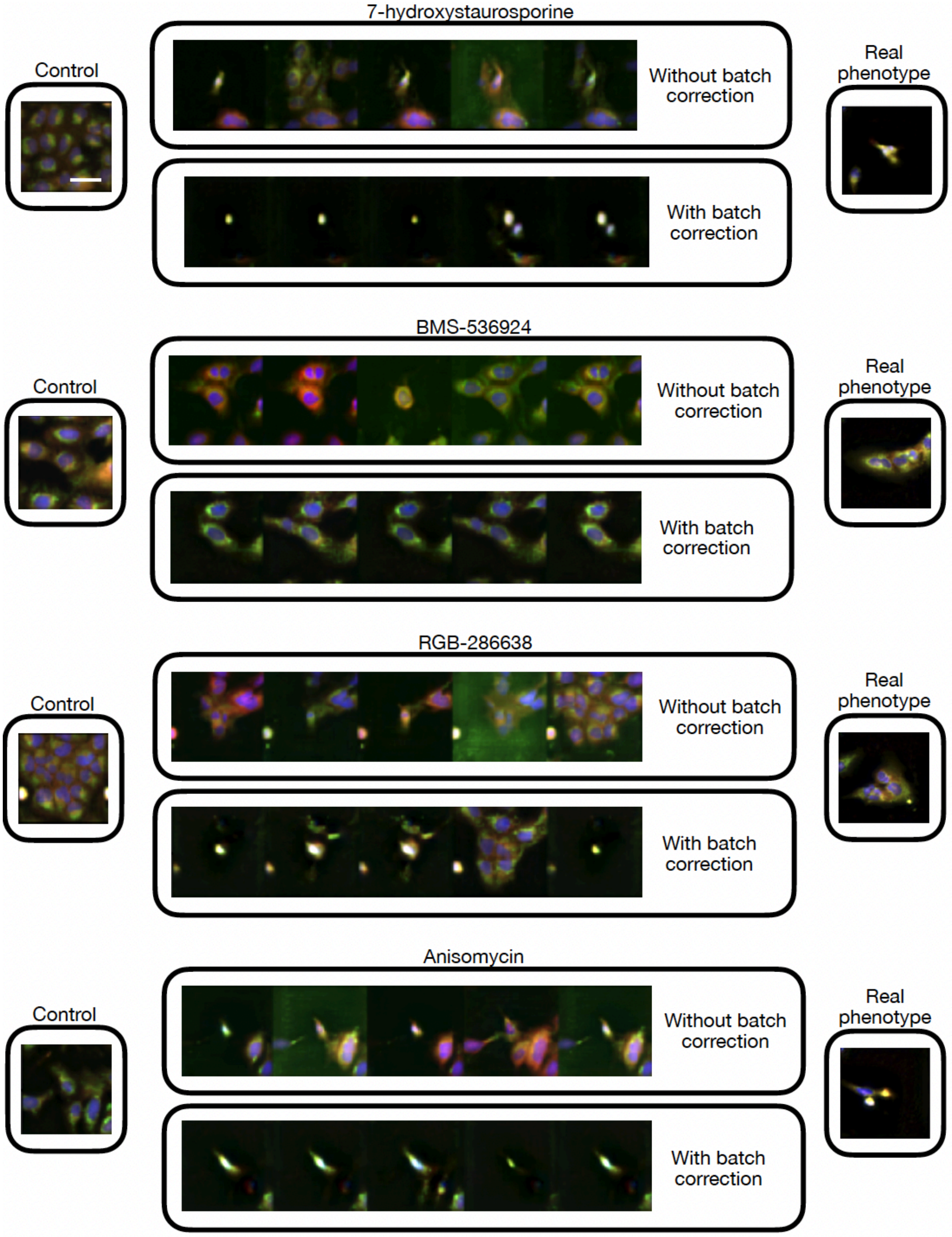

Supplementary Figure 9 | Additional examples of the importance of correcting for a batch for learning perturbation predictions. The top row in each box shows the predictions of the effect of a perturbation without performing plate correction. The bottom row illustrates the results after plate correction. On the right, is an example of an expected phenotype. The scale bar is 30 µm

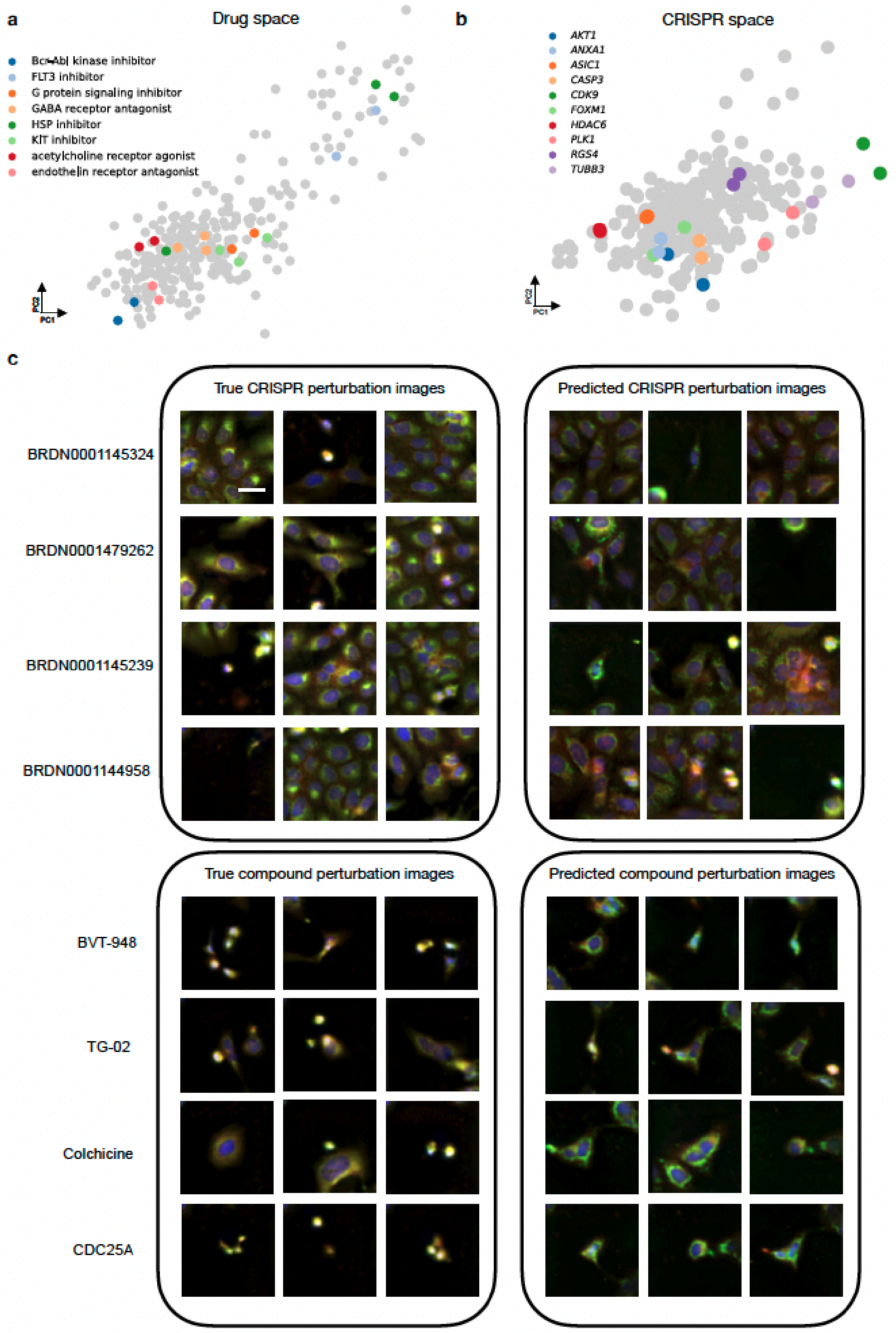

Supplementary Figure 10 | Additional results on the cpg0000 dataset. (a) The PCA space of the representation of drug perturbations learnt by IMPA annotated by the compound name. **(b)** The PCA space of the representation of CRISPR perturbations learnt by IMPA annotated by the target gene name. **(c)** Examples of predictions and real perturbation images of five unseen CRISPR and compound treatments in cpg0000. Multiple examples are included both in the predictions and real images to show how more penetrant the phenotypic effect of compounds is compared to CRISPR perturbations and how the predictions from our model are capable of capturing such a behavior. The scale bar is 30 μm.

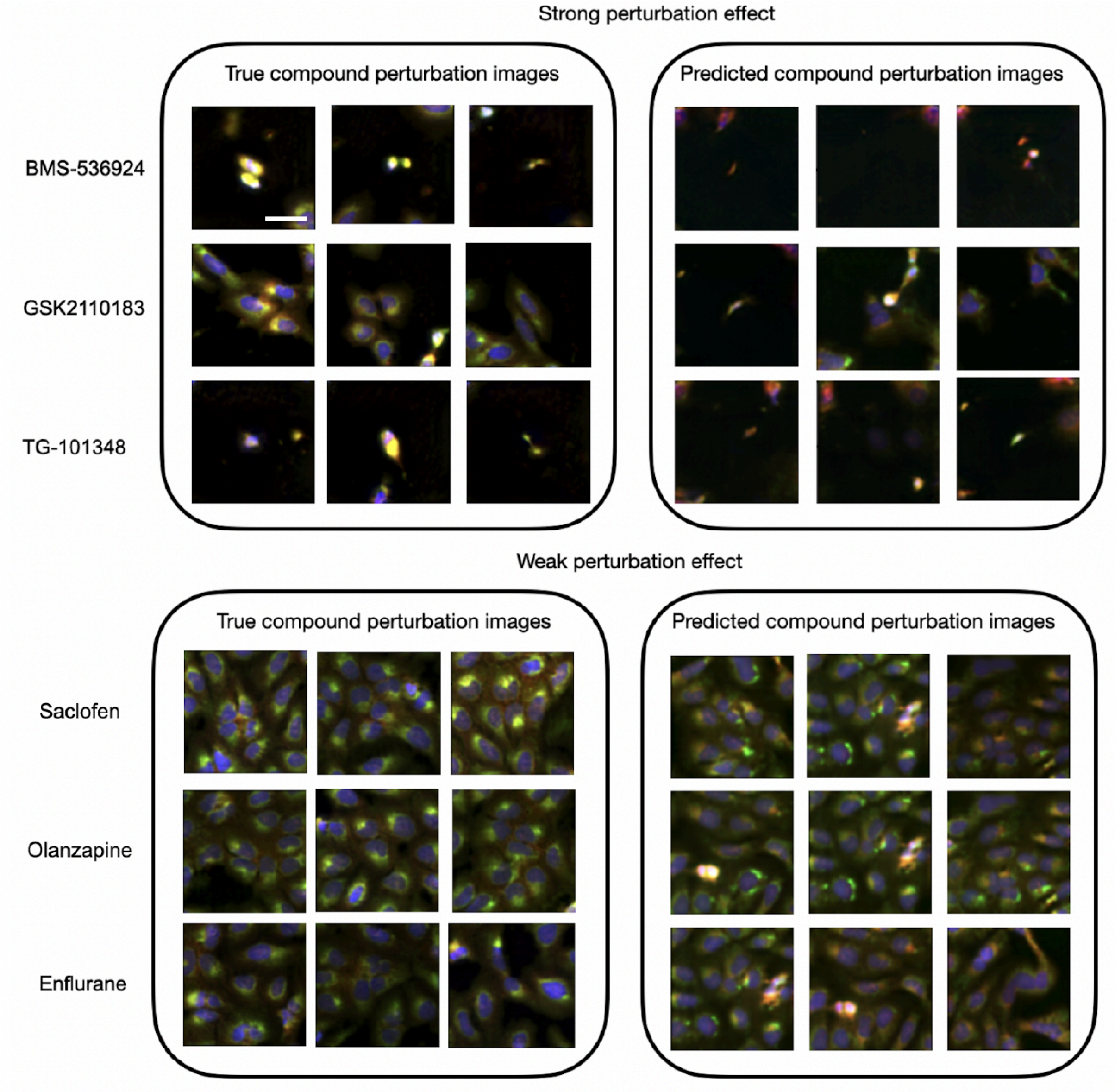

Supplementary Figure 11 | Additional predictions on cpg0000. Prediction of strong and weak perturbation effects by IMPA. The scale bar is 30 μm.

Tables

Supplementary Table 1 | BBBC021 compounds and the associated dosages considered in this work.

**Compound Name**	**Non-zero Dosages (μM)**
5-fluorouracil	0.003,0.01,0.03,0.1,0.3,1.0,3.0,10.0
Acyclovir	0.0015,0.005,0.015,0.05,0.15,0.5,1.5,5.0
AG-1478	0.003,0.006,0.01,0.02,0.03,0.06,0.1,0.2,0.3,0.6,1.0,2.0,3.0,6.0,10.0,20.0
ALLN	0.03,0.1,0.3,1.0,10.0,30.0,3.0,100.0
Aloisine A	0.01,0.03,0.1,0.3,1.0,3.0,10.0,30.0
Alsterpaullone	0.01,0.03,0.1,0.3,10.0,30.0,1.0,3.0
Anisomycin	0.003,0.01,0.03,0.1,3.0,10.0,0.3,1.0
Aphidicolin	0.003,0.01,0.03,0.1,0.3,1.0,3.0,10.0
Arabinofuranosylcytosine	0.01,0.03,0.1,0.3,1.0,3.0,10.0,30.0
Atropine	0.0015,0.005,0.015,0.05,0.15,0.5,1.5,5.0
Bleomycin	0.015,0.05,0.15,0.5,1.5,5.0,15.0,50.0
Bohemine	0.006,0.02,0.06,0.2,0.6,2.0,6.0,20.0
Brefeldin A	0.003,0.01,0.03,0.1,0.3,1.0,3.0,10.0
Bryostatin	0.003,0.01,0.03,0.1,1.0,3.0,10.0,0.3
Calpain inhibitor 2 (ALLM)	0.015,0.05,0.15,0.5,1.5,5.0,15.0,50.0
Calpeptin	0.015,0.05,0.15,0.5,1.5,5.0,15.0,50.0
Camptothecin	0.1,0.3,1.0,3.0,10.0,0.003,0.01,0.03
Carboplatin	0.006,0.02,0.06,0.2,0.6,2.0,6.0,20.0
Caspase inhibitor 1 (ZVAD)	0.015,0.05,0.15,0.5,1.5,5.0,15.0,50.0
Cathepsin inhibitor I	0.003,0.01,0.03,0.1,0.3,1.0,3.0,10.0
Cdk1 inhibitor III	0.003,0.01,0.03,0.1,0.3,1.0,3.0,10.0
Cdk1/2 inhibitor (NU6102)	0.003,0.01,0.03,0.1,0.3,1.0,3.0,10.0
Chlorambucil	0.003,0.01,0.03,0.1,0.3,1.0,3.0,10.0
Chloramphenicol	0.006,0.02,0.06,0.2,0.6,2.0,6.0,20.0
Cisplatin	0.01,0.03,0.1,0.3,1.0,3.0,30.0,10.0
Colchicine	0.001,0.003,0.01,0.1,0.3,1.0,3.0,0.03
Cyclohexamide	0.015,0.05,0.15,0.5,1.5,5.0,15.0,50.0
Cyclophosphamide	0.0015,0.005,0.015,0.05,0.15,0.5,1.5,5.0
Cytochalasin B	0.01,0.03,0.1,0.3,1.0,3.0,10.0,30.0
Cytochalasin D	0.003,0.01,0.03,0.1,1.0,3.0,10.0,0.3
Demecolcine	0.003,0.01,0.03,0.1,0.3,1.0,3.0,10.0
Deoxymannojirimycin	0.3,1.0,3.0,10.0,30.0,100.0,300.0,1000.0
Deoxynojirimycin	0.3,1.0,3.0,10.0,30.0,100.0,300.0,1000.0
3,3'-diaminobenzidine	0.003,0.01,0.03,0.1,0.3,1.0,3.0,10.0
Docetaxel	0.0003,0.001,0.003,0.01,1.0,0.03,0.1,0.3
Doxorubicin	0.003,0.01,0.03,0.1,0.3,1.0,3.0,10.0
Emetine	0.01,0.03,3.0,10.0,30.0,0.1,0.3,1.0

Epothilone B	0.001,0.003,0.01,0.03,3.0,0.1,0.3,1.0
Etoposide	0.01,0.03,0.1,0.3,30.0,1.0,3.0,10.0
Filipin	0.003,0.01,0.03,0.1,0.3,1.0,3.0,10.0
Floxuridine	0.03,0.1,0.3,1.0,3.0,100.0,10.0,30.0
Forskolin	0.01,0.03,0.1,0.3,1.0,3.0,10.0,30.0
Genistein	0.03,0.1,0.3,1.0,3.0,10.0,30.0,100.0
H-7	0.015,0.05,0.15,0.5,1.5,5.0,15.0,50.0
Herbimycin A	0.003,0.01,0.03,0.1,0.3,1.0,3.0,10.0
Hydroxyurea	0.3,1.0,3.0,10.0,30.0,100.0,300.0,1000.0
ICI-182,780	0.01,0.03,0.1,0.3,1.0,3.0,10.0,30.0
Indirubin monoxime	0.003,0.01,0.03,0.1,0.3,1.0,3.0,10.0
Jasplakinolide	0.0003,0.001,0.003,0.01,0.03,0.1,0.3,1.0
Lactacystin	0.003,0.01,0.03,0.1,0.3,1.0,3.0,10.0
Latrunculin B	0.01,0.03,0.1,0.3,10.0,30.0,1.0,3.0
Leupeptin	0.006,0.02,0.06,0.2,0.6,2.0,6.0,20.0
LY-294002	0.0006,0.002,0.006,0.02,0.06,0.2,0.6,2.0
Methotrexate	0.003,0.01,0.03,0.1,0.3,1.0,3.0,10.0
Methoxylamine	0.015,0.05,0.15,0.5,1.5,5.0,15.0,50.0
Mevinolin/lovastatin	0.015,0.05,0.15,0.5,50.0,1.5,5.0,15.0
MG-132	0.03,0.3,1.0,10.0,30.0,100.0,0.1,3.0
Mitomycin C	0.003,0.01,0.03,10.0,0.1,0.3,1.0,3.0
Mitoxantrone	0.03,0.1,0.3,1.0,3.0,10.0,0.003,0.01
Monastrol	0.01,0.03,0.1,0.3,1.0,3.0,10.0,30.0,100.0
Neomycin	0.003,0.01,0.03,0.1,0.3,1.0,3.0,10.0
Nocodazole	0.001,0.003,0.01,0.03,0.1,0.3,1.0,3.0
Nystatin	0.003,0.01,0.03,0.1,0.3,1.0,3.0,10.0
Okadaic acid	6e-05,0.0002,0.0006,0.002,0.006,0.02,0.06,0.2
Olomoucine	0.003,0.01,0.03,0.1,0.3,1.0,3.0,10.0
PD-150606	0.015,0.05,0.15,0.5,1.5,5.0,15.0,50.0
PD-169316	0.01,0.03,0.1,0.3,1.0,30.0,3.0,10.0
PD-98059	0.01,0.03,0.1,0.3,1.0,3.0,10.0,30.0
Podophyllotoxin	3e-06,1e-05,3e-05,0.0001,0.0003,0.001,0.003,0.01
PP-2	0.01,0.03,0.1,0.3,1.0,30.0,3.0,10.0
Proteasome inhibitor I	0.003,0.01,0.03,0.3,1.0,10.0,0.1,3.0
Puromycin	0.03,0.1,0.3,1.0,3.0,10.0,30.0,100.0
Quercetin	0.003,0.01,0.03,0.1,0.3,1.0,3.0,10.0
Raloxifene	0.0015,0.005,0.015,0.05,0.15,0.5,1.5,5.0
Rapamycin	0.003,0.01,0.03,0.1,0.3,1.0,3.0,10.0
Roscovitine	0.003,0.01,0.03,0.1,0.3,1.0,3.0,10.0
SB-202190	0.006,0.02,0.06,0.2,0.6,2.0,6.0,20.0
SB-203580	0.003,0.01,0.03,0.1,0.3,1.0,3.0,10.0
Simvastatin	0.006,0.02,0.06,0.2,0.6,2.0,6.0,20.0
Sodium butyrate	0.083,0.25,0.83,2.5,8.3,25.0,83.0,250.0

Sodium fluoride	0.15,0.5,1.5,5.0,15.0,50.0,150.0,500.0
SP-600125	0.015,0.05,0.15,0.5,1.5,5.0,15.0,50.0
Staurosporine	0.0003,0.001,0.003,0.01,0.03,0.1,0.3,1.0
Taurocholate	0.0083,0.025,0.083,0.25,0.83,2.5,8.3,25.0
Taxol	0.001,0.003,0.01,0.03,0.1,0.3,1.0,3.0
Temozolomide	0.006,0.02,0.06,0.2,0.6,2.0,6.0,20.0
Trichostatin	0.0003,0.001,0.003,0.01,0.03,0.1,0.3,1.0
Tunicamycin	0.015,0.05,0.15,0.5,1.5,5.0,15.0,50.0
UO-126	0.01,0.03,0.1,0.3,1.0,3.0,10.0,30.0
Valproic acid	0.15,0.5,1.5,5.0,15.0,50.0,150.0,500.0
Vinblastine	0.003,0.01,0.03,0.1,0.3,1.0,3.0,10.0
Vincristine	10.0,0.003,0.01,0.03,0.1,0.3,1.0,3.0
Y-27632	0.01,0.03,0.1,0.3,1.0,3.0,10.0,30.0
AZ235	0.01,0.03,0.1,0.3,1.0,3.0,10.0,30.0
AZ138	0.01,10.0,30.0,0.03,0.1,0.3,1.0,3.0
AZ701	0.01,0.03,0.1,0.3,1.0,3.0,10.0,30.0
AZ258	0.01,0.03,3.0,10.0,30.0,0.1,0.3,1.0
AZ841	0.01,0.03,3.0,10.0,30.0,0.1,0.3,1.0
DMSO	0.0

Supplementary Table 2 | Time (in seconds) for generation with IMPA and PhenDiff as a function of the batch size.

	**Batch size 8**	**Batch size 16**	**Batch size 32**	**Batch size 64**	**Batch size 128**
PhenDiff	23.58	28.21	55.25	107.90	220.62
IMPA	0.03	0.08	0.08	0.08	0.09

Supplementary Table 3 | Couples of closest compounds and their Tanimoto similarity in BBBC021.

Reference compound	Closest compound	Tanimoto Similarity
5-fluorouracil	Floxuridine	0.27
Acyclovir	Olomoucine	0.21
AG-1478	AZ701	0.33
ALLN	Calpain inhibitor 2 (ALLM)	0.82
Aloisine A	SB-202190	0.27
Alsterpaullone	Indirubin monoxime	0.35
Anisomycin	PD-150606	0.26
Aphidicolin	Taurocholate	0.24
Arabinofuranosylcytosine	Puromycin	0.33
Atropine	Y-27632	0.22
Bleomycin	Tunicamycin	0.20
Bohemine	Roscovitine	0.74
Brefeldin A	Latrunculin B	0.26
Bryostatin	Docetaxel	0.23
Calpain inhibitor 2 (ALLM)	ALLN	0.82
Calpeptin	MG-132	0.62
Camptothecin	Cdk1 inhibitor III	0.24
Carboplatin	Latrunculin B	0.13
Caspase inhibitor 1 (ZVAD)	Proteasome inhibitor I	0.51
Cdk1 inhibitor III	PD-98059	0.34
Cdk1/2 inhibitor (NU6102)	AZ235	0.31
Chlorambucil	PD-150606	0.28
Chloramphenicol	Alsterpaullone	0.21
Cisplatin	Methoxylamine	0.07
Colchicine	Demecolcine	0.85
Cyclohexamide	Mevinolin/lovastatin	0.20
Cyclophosphamide	Chlorambucil	0.21
Cytochalasin B	Cytochalasin D	0.54
Cytochalasin D	Cytochalasin B	0.54
Demecolcine	Colchicine	0.85
Deoxymannojirimycin	Deoxynojirimycin	1.00
Deoxynojirimycin	Deoxynojirimycin	1.00
3,3'-diaminobenzidine	Genistein	0.27
Docetaxel	Taxol	0.73
Doxorubicin	Etoposide	0.22
Emetine	Demecolcine	0.32
Epothilone B	Forskolin	0.25
Etoposide	Podophyllotoxin	0.57
Filipin	Nystatin	0.33
Floxuridine	Arabinofuranosylcytosine	0.28
Forskolin	Epothilone B	0.25

Genistein	Quercetin	0.49
H-7	AZ235	0.21
Herbimycin A	Rapamycin	0.26
Hydroxyurea	Sodium butyrate	0.18
ICI-182,780	Taurocholate	0.17
Indirubin monoxime	SP-600125	0.35
Jasplakinolide	Cytochalasin D	0.23
Lactacystin	Calpain inhibitor 2 (ALLM)	0.26
Latrunculin B	Brefeldin A	0.26
Leupeptin	ALLN	0.79
LY-294002	PD-98059	0.39
Methotrexate	Trichostatin	0.24
Methoxylamine	DMSO	0.08
Mevinolin/lovastatin	Simvastatin	0.78
MG-132	Calpeptin	0.62
Mitomycin C	Herbimycin A	0.19
Mitoxantrone	Quercetin	0.22
Monastrol	Cdk1 inhibitor III	0.30
Neomycin	Tunicamycin	0.28
Nocodazole	Alsterpaullone	0.22
Nystatin	Filipin	0.33
Okadaic acid	Rapamycin	0.20
Olomoucine	Bohemine	0.72
PD-150606	Chlorambucil	0.28
PD-169316	SB-202190	0.80
PD-98059	LY-294002	0.39
Podophyllotoxin	Etoposide	0.57
PP-2	AG-1478	0.25
Proteasome inhibitor I	MG-132	0.58
Puromycin	Arabinofuranosylcytosine	0.33
Quercetin	Genistein	0.49
Raloxifene	SB-202190	0.28
Rapamycin	Herbimycin A	0.26
Roscovitine	Bohemine	0.74
SB-202190	PD-169316	0.80
SB-203580	SB-202190	0.80
Simvastatin	Mevinolin/lovastatin	0.78
Sodium butyrate	Valproic acid	0.32
Sodium fluoride	5-fluorouracil	0.00
SP-600125	PD-98059	0.35
Staurosporine	Indirubin monoxime	0.27
Taurocholate	Aphidicolin	0.24
Taxol	Docetaxel	0.73

Temozolomide	Acyclovir	0.17
Trichostatin	Methotrexate	0.24
Tunicamycin	Neomycin	0.28
UO-126	3,3'-diaminobenzidine	0.23
Valproic acid	Sodium butyrate	0.32
Vinblastine	Vincristine	0.91
Vincristine	Vinblastine	0.91
Y-27632	Atropine	0.22
AZ235	Cdk1/2 inhibitor (NU6102)	0.31
AZ138	AZ701	0.25
AZ701	AG-1478	0.33
AZ258	AZ841	0.90
AZ841	AZ258	0.90
DMSO	Valproic acid	0.10
