# Supplementary material for: Predicting cell morphological responses to perturbations using generative modeling
Source: Nat Commun. 2025 Jan 8;16:505. doi: 10.1038/s41467-024-55707-8 (PMC11711326; doi:10.1038/s41467-024-55707-8)
Supplement: Supplementary file 3 — Peer Review file [file 41467_2024_55707_MOESM3_ESM.pdf]

nature portfolio

Peer Review File

Predicting cell morphological responses to perturbations using generative modeling

Corresponding Author: Dr Mohammad Lotfollahi

This manuscript has been previously reviewed at another journal. This document only contains information relating to versions considered at Nature Communications.

This file contains all reviewer reports in order by version, followed by all author rebuttals in order by version.

Version 0:

Reviewer comments:

Reviewer #2

(Remarks to the Author)
Authors have addressed major concerns raised by reviewer 2 and 3 in the revision.

(Remarks on code availability)
The code is functional.

Reviewer #3

(Remarks to the Author)
The paper develops an IMage Perturbation Autoencoder (IMPA), a generative style-transfer model to predict morphological changes of perturbations across both genetic and chemical interventions absent from the training data.

I checked all the materials - The authors have sufficiently addressed my questions.

(Remarks on code availability)
The code is provided with detailed documentations.

Open Access This Peer Review File is licensed under a Creative Commons Attribution 4.0 International License, which permits use, sharing, adaptation, distribution and reproduction in any medium or format, as long as you give appropriate credit to the original author(s) and the source, provide a link to the Creative Commons license, and indicate if changes were made.
In cases where reviewers are anonymous, credit should be given to 'Anonymous Referee' and the source.
The images or other third party material in this Peer Review File are included in the article's Creative Commons license, unless indicated otherwise in a credit line to the material. If material is not included in the article's Creative Commons license and your intended use is not permitted by statutory regulation or exceeds the permitted use, you will need to obtain permission directly from the copyright holder.
To view a copy of this license, visit https://creativecommons.org/licenses/by/4.0/
